# Isolation, characterization and analysis of bacteriophages from the haloalkaline lake Elmenteita, Kenya

**DOI:** 10.1371/journal.pone.0215734

**Published:** 2019-04-25

**Authors:** Juliah Khayeli Akhwale, Manfred Rohde, Christine Rohde, Boyke Bunk, Cathrin Spröer, Hamadi Iddi Boga, Hans-Peter Klenk, Johannes Wittmann

**Affiliations:** 1 Leibniz Institute DSMZ – German Collection of Microorganisms and Cell Cultures, Braunschweig, Germany; 2 Department of Zoology, Jomo Kenyatta University of Agriculture and Technology, Nairobi, Kenya; 3 Helmholtz Centre for Infection Research, Central Facility for Microscopy, Braunschweig, Germany; 4 Taita Taveta University College, Voi, Kenya; 5 School of Natural and Environmental Sciences, Newcastle University, Newcastle upon Tyne, United Kingdom; Louisiana State University, UNITED STATES

## Abstract

As a step towards better understanding of diversity and biology of phages and their hosts in haloalkaline Lake Elmenteita, phages were isolated from sediment samples and overlying water using indigenous bacteria as hosts. 17 seemingly different phages of diverse morphotypes with different dimensions and partly exhibiting remarkably unusual ultrastructures were revealed by transmission electron microscopy. 12 clonal phage isolates were further characterized. Infection capability of the phages was optimum at 30–35°C and in alkali condition with optimum at pH 10–12. Structural protein profiles and restriction fragment length polymorphism analyses patterns were distinct for each of the phage type. Complete nucleotide sequences of phages vB-VmeM-32, vB_EauS-123 and vB_BhaS-171 genomes varied in size from 30,926–199,912 bp and G + C content of between 36.25–47.73%. A range of 56–260 potential open reading frames were identified and annotated. The results showed that the 12 phages were distinct from each other and confirmed the presence and diversity of phages in extreme environment of haloalkaline Lake Elmenteita. The phages were deposited at the German Collection of Microorganisms and Cell Cultures and three of their genomes uploaded to NCBI GenBank.

## Introduction

Viruses that infect bacteria called bacteriophages (commonly referred to as phages) are known to exist in essentially every possible niche where bacteria reside [1] and profoundly influence ecosystems by infecting and subsequently killing their hosts, thereby impacting the cycling of carbon and nutrients [2]. In environments of extreme temperature, pH, salinity, or a combination of these conditions, viruses of archaea are well represented [3]. In these extreme environments, viruses are the only known predators of prokaryotes. Virus particles in hot springs have been observed by electron microscopy [4] and also cultured on bacteria and archaea isolated from these ecosystems [5][6][7][8]. Witte et al [9] isolated a novel archaeal virus, ɸCh1 from a haloalkalophilic archaeon *Natronobacterium magadii* upon spontaneous lysis. Danovaro et al [10] evaluated the selectivity of viral infections on deepsea floor by using several independent approaches, including an innovative molecular method based on the quantification of archaeal versus bacterial genes released by viral lysis. Since many viruses are strain-specific, when a particular microbial strain becomes dominant in a system, the number of its viral predators increases exponentially and kill it off leaving a niche for another microbial strain to grow into, that will subsequently be killed off by another viral type. This “kill-the-winner” hypothesis explains much of the observed microbial diversity and changes in community structure [11].

Natural phage communities are reservoirs of considerable uncharacterized genetic diversity on Earth [12] and provide a valuable resource to development of modern biotechnology [13]. Complete phage genomes facilitate studies of phage evolutionary history and relationships, biodiversity, biogeography and identification of novel phage taxa [14][15][16]. Insight into understanding of phage biology can be exploited to generate a broad application spectrum like novel nanotechnologies, bacterial detection strategies and biological control of pathogenic bacteria on an industrial scale [17][18]. Despite their importance and ubiquitous abundance, far too little is known about their diversity in natural ecosystems [19][20][21][22]. The best studied groups of phages are those examples infecting bacterial pathogens. Most studies on natural populations of phages and their host relationship have been performed in terrestrial, marine, and freshwater environments and very few in unusual or extreme habitats [23][24][25] where over 90% of the earth’s bacterial diversity is thought to reside [26][25]. Furthermore, most known phages are from North America and Europe, while little is known of phages in the environment of vast regions such as Africa and South America [27].

Soda lakes are strongly alkaline lakes, typically with a pH of 8.5 to >12, high concentrations of carbonate ions and with salinities ranging from brackish to hypersaline [28]. The groups of microbes able to grow under alkaline conditions in the presence of high salt are referred to as haloalkaliphiles [29]. They possess special adaptation mechanisms to survive and grow under high salinity and alkaline pH. These properties of dual extremity of halophiles and alkaliphiles make them interesting from both fundamental research and biotechnological points of view [30]. Kenya's Great Rift Valley contains this type of Lakes namely Elmenteita, Magadi, Bogoria, Nakuru and Sonachi [31]. Studies on diversity and isolation of bacterial species from Lake Elmenteita have been highly documented [32][33][34]. However, viruses from these environments are particularly under-studied at present. Hence, rich reservoirs of enormous genetic and biological diversity therefore remain to be explored and analyzed. Previous studies on the Soda lakes include isolation of phages from Lake Magadi by Jamison et al [35] and Muruga et al [36]. Moulton et al [37] also isolated and studied a phage infecting an alkaliphilic *Vibrio metschnikovii* from Lake Magadi. Peduzzi et al [38] carried out an electron microscopic study of cyanophages that affect African flamingo population in Lake Nakuru.

As a step towards better understanding of the diversity and biology of phages and their hosts in haloalkaline Lake Elmenteita, phages were isolated from sediments and overlying water using indigenous bacteria as hosts. The phages were characterized by their morphology, host range analysis, structural protein profile analysis, restriction endonuclease patterns analysis and genome size estimation by pulsed-field gel electrophoresis (PFGE). A further goal of this research was to sequence, annotate and analyse the genome of some phages from the haloalkaline Lake Elmenteita using various available bioinformatics tools.

The study site, Lake Elmenteita, is situated at 0° 27ʹ S 36° 15ʹ E on the floor of the Kenyan Rift Valley at 1776 m above sea level and has no direct outlet [39]. The region is characterized by a hot, dry and semi-arid climate with a mean annual rainfall of about 700 mm [40]. Due to the high temperatures there are very high evaporation rates during the drier seasons, leading to a seasonal reduction in the total surface area. The size of Lake Elmenteita is roughly 20 km^2^ and the depth rarely exceeds 1.0 m [33]. The alkalinity of the water is high with a high concentration of carbonates (1200 mg Na_2_CO_3_ l^-1^), chlorides and sulphates [32]. The water temperature ranges between 30 and 40°C and the pH is above 9.

It is expected that the genomic sequences will give insight into genome architecture and content in terms of gene function as well as the level of their similarity compared to currently available phages. To our knowledge, these experiments represent the first report of isolation and characterization of bacteriophages from the haloalkaline Lake Elmenteita.

## Materials and methods

Research authorization in Kenya was given by the National Commission for Science, Technology and Innovation (NACOSTI), Kenya Wildlife Service (KWS) and National Environmental Management Authority (NEMA).

### Isolation and characterization of bacterial host strains

Sediment sample plus the overlying water were collected (March, 2013) into sterile jars, capped on site and preserved in cooled boxes for transportation to the molecular laboratory in Jomo Kenyatta University of Agriculture and Technology (JKUAT). In the laboratory the samples were packaged for transfer to Leibniz Institute—DSMZ in Braunschweig, Germany and stored at 8°C.

Approximately 2 g of sediment was used to make a mastermix using filter (0.20 μm pore size) sterilised water (10 ml) from the lake and the solution serially diluted using the same water. Aliquots (100 μl) of serial dilutions were plated onto solid LB medium adjusted to approximately pH 9.5 with Sodium Sesquicarbonate (4.3 g NaHCO_3_/5.2 g NaCO_3_/100 ml distilled water). The plates were incubated at 28°C for 3 days. Colonies appearing on the plates were purified by three consecutive single colony passages. Isolated bacterial strains were used as hosts for the detection of lytic bacteriophages from the same lake. Susceptible strains were stocked in LB broth (pH 9.5) with 15% glycerol (v/v) at -20°C.

Growth of the strains on different media (LB, Nutrient agar and Horikoshi 1) at 28°C was assessed. Growth was also assessed at temperature 20–45°C (in increments of 5°C), pH values from 5.0–13.0 (in increments of 1.0 pH unit) using LB as the basal medium. The colony features were observed under a binocular microscope [41]. Cell morphology (size, shape, arrangement) was determined by phase-contrast microscopy (magnification, 400×) after 3 days of incubation at 28°C. Gram stain was performed using the KOH test [42]. Bacterial hosts’ genomic DNA extraction, PCR-mediated amplification of 16S rDNA gene using universal bacterial primer sets 27F (5’-AGAGTTTGATCMTGGCTCAG-3’) and 1492R (5’-TACGGYTACCTTGTTACGACTT-3’), and purification of PCR products was carried out as previously described by Kim et al [43]. Identification of phylogenetically closest taxa and calculation of pairwise 16S rRNA gene sequence similarity was performed using the EzTaxon server (http://www.eztaxon.org) [44]. The genomic homogeneity of the strains was also examined in comparison with their close relatives by Matrix assisted laser desorption/ionization time of flight (MALDI-TOF) mass spectra (MS) analysis [45].

### Isolation and characterization of bacteriophages

#### Bacteriophage propagation and purification

LB medium supplemented with 2mM CaCl_2_ (Sigma-Aldrich, St. Louis, MO) adjusted to approximately pH 9.5 using 1M Sodium-Sesquicarbonate (4.3 g NaHCO_3_, 5.2 g NaCO_3_, 100 ml distilled water; 1 ml in 10 ml medium) was used. Approximately 1 g of sediment sample was suspended in 9 ml LB broth in a sterile 15 ml centrifuge tube and mixed thoroughly on an overhead shaker for 1 hour at room temperature. The sample was thereafter centrifuged at 7,500 r.p.m. for 15 minutes then the supernatant further filtered through a 0.45 μm pore size syringe filter (Millipore corp, Billerica, MA). The supernatant (5 ml) was added to equal amount of double strength LB broth and inoculated with an early log-phase (0.1 ml) host culture. After overnight enrichment at 28°C with gentle shaking, the culture was centrifuged at 7,500 r.p.m. for 15 minutes [46]. This enrichment procedure was repeated thrice. The supernatant obtained from the final enrichment step was filter sterilized through a 0.45 μm pore size syringe filter and checked for the presence of phages by the soft agar overlay method. The soft agar was prepared by adding 100 μl phage lysate to 200 μl of an overnight culture of indicator strain and mixed with 5 ml of liquid soft agar at 45°C. This mixture was spread on solid LB medium, incubated overnight at 28°C and checked for the presence of plaques [47]. Uninfected host strain was used as negative control for checking bacteriocin reactions to confirm the validity of plaques [48]. Underlay procedure for phage purification [46] was followed. Phage particle from a well isolated plaque was streaked on solid LB medium as though attempting to obtain single colony isolates from a bacterial culture, followed by overlay containing host cells poured over the surface of the plate and incubated after setting. The procedure was repeated three times.

To recover phages, phages were collected from plates with confluent lysis and eluted by transferring agar overlayer aseptically into 10 ml of mid-log host cell culture in LB broth and incubated at 28°C with gentle shaking (overnight). The phage supernatant was collected by centrifugation at 7,500 r.p.m. for 15 minutes, filtered (0.45 μm) and the phage stock stored at 4°C. The titer of the stock was determined by the soft agar overlay method. 1 ml of the phage lysate was transferred aseptically to 10 ml of the mid-log host cell culture in LB broth and incubated at 28°C with gentle shaking until clearing was observed (overnight). The phage supernatant was collected by centrifugation at 7,500 r.p.m. for 15 minutes (Sorvall RC6, F10S-6×500y rotor). The fresh lysate (10 ml) was added to 200 ml of mid-log host cell culture and repeated as above. Phages were concentrated by centrifugation at 12,000 r.p.m. for 2 hours (Sorvall RC6, F21S-8 × 50 rotor). Phages were purified using CsCl density-gradient ultracentifugation. The phage pellet was re-suspended in 1 ml of TE buffer (20 mM Tris [pH 7.5], 50 mM NaCl) [49]. 1.5 ml concentrated phage suspension was overlaid onto a four-step Cesium Chloride (CsCl) gradient containing 0.7 ml each of 1.7 g/ml, 1.5 g/ml, 1.4 g/ml and 1.3 g/ml CsCl (Optical grade, Gibco) in a 4.3 ml ultracentrifuge tube (Beckman Coulter). Phages were centrifuged for 2 h at 20°C and 22,000 r.p.m. in ultracentrifuge (Beckman Coulter, Optima L-XP; SW 60 Ti 12E873 rotor). Phage-containing bands (white-to-grey) were extracted by puncturing the wall of the ultracentrifuge tube using a needle, and the CsCl removed by dialysis (visking dialysis tubing: Type (inch) 8/32, wall thickness (mm) 0.050, width (mm) 10, Ø (mm) 6.3; ROTH) for 15 h with two changes of TE buffer (10 mM Tris [pH 7.5], 50 mM NaCl) [49].

#### Negative staining and electron microscopy of bacteriophages

Thin carbon support films were prepared by sublimation of a carbon thread onto a freshly cleaved mica surface. Phages were adsorbed onto the carbon film and negatively stained with 2% (w/v) aqueous uranyl acetate, pH 5.0 [50]. Samples were examined in a TEM 910 transmission electron microscope (Carl Zeiss, Oberkochen) at an acceleration voltage of 80 kV. Images were taken at calibrated magnifications using a line replica. Images were recorded digitally with a Slow-Scan CCD-Camera (ProScan, 1024×1024, Scheuring, Germany) with ITEM-Software (Olympus Soft Imaging Solutions, Münster, Germany). The phenotypic diversity of the bacteriophages was determined using the morphological criteria outlined by the International Committee of Taxonomy of Viruses [27].

#### Thermal and pH stability tests

The thermal stability of the phages was examined by pre-incubating phage suspensions at different temperatures (20, 25, 30, 35, 40, 45 and 50°C respectively) at pH 7.0 for 6 hours. After the incubation, phage suspensions were immediately cooled in ice water and the surviving phages were tittered by the double agar layer method. The pH stability of phages was examined by pre-incubating the phage suspensions of different pH levels (2, 4, 6, 8, 10 and 12 respectively) at 25°C for 6 hours. The surviving phages were immediately counted by the double agar layer method [51].

#### Host range analysis of bacteriophages

To evaluate the lytic spectrum of the obtained bacteriophages, all the susceptible bacterial strains isolated in this study were used. Double layer agar plates with different bacterial strains were prepared. The lysis spectrum of isolated phages was determined by spotting 10 μl of phage lysate on each agar plate with different bacterial strains. The plates were incubated at 28°C overnight and examined for clearing zones [52]. Observed inhibition of growth as marked by clearing where the lysate was spotted, was denoted as susceptibility of the bacteria.

#### Stuctural protein profiles

Sodium Dodecyl Sulfate-Polyacrylamide Gel Electrophoresis (SDS-PAGE) was performed by the method of Laemmli [53]. A sample of 50 μl purified phage particles (5×10^10^ pfu/ml) was dissolved in 50 μl loading buffer (50 μl Mercaptoethanol, 950 μl Laemmli sample buffer (2×) for SDS-PAGE; SERVA electrophoresis). After heating at 95°C for 5 min, the samples were subjected to electrophoresis in 12% SDS-PAGE gel along with protein marker (PageRuler Broad Range Unstained protein ladder; Thermo Scientific) with Tris-glycine as running buffer. After electrophoresis, proteins were visualized by staining with Coomassie Brilliant Blue R250 dye (Sigma).

### Bacteriophage genomic and phylogenetic analysis

#### DNA extraction

DNA was extracted from CsCl purified high-titre stocks of phage using phage DNA isolation kit (Norgen Biotek Corp., Thorold, ON, Canada) according to the manufacturer’s instructions. The purity and the concentration of the DNA were determined using spectrophotometer (Invitrogen Qubit).

#### Genome estimation

Pulsed field gel electrophoresis (PFGE) was used to estimate the genomes sizes for the 14 phage isolates according to the protocol published by Lingohr et al [54]. Plugs were prepared according to procedure and gel run at 5 V/cm for 24 h at 14°C with initial switch at 5s and final switch 15s.

#### Restriction digestion patterns

For comparison of DNA fragment patterns, phage genomic DNA was digested with different restriction endonucleases according to the instructions of the manufacturer (Fermentas life sciences, UK). A total of five restriction endonucleases namely; *Dra*I, *Eco*RI, *Hind*III, *Kpn*I and *Pst*I were used. Restriction fragments were separated by electrophoresis (1h, 90V) on 1.0% agarose (Sigma, USA) gel stained with ethidium bromide. DNA molecular weight marker (mi-1Kb DNA Marker; Metabion, Germany) was used for size determination of DNA fragments [55].

#### PacBio library preparation and sequencing

Three bacteriophages of this study; vB-VmeM-32, vB_EauS-123 and vB_BhaS-171, were randomly chosen for complete genome study. SMRTbell template libraries were prepared according to the instructions from Pacific Biosciences, Menlo Park, CA, USA, following the Procedure and Checklist Greater than 10 kb Template Preparation and Sequencing using a multiplex workflow with symmetric barcoded adapter of 16 nucleotides (F1 to F3), each for one of the phages. Briefly, for preparation of 10kb libraries ~ 4μg genomic DNA isolated from phages were sheared applying g-tubes from Covaris (Woburn, MA) according to the manufacturer´s instructions. DNAs were end-repaired and ligated overnight to hairpin adapters applying components from the DNA/Polymerase Binding Kit P5 from Pacific Biosciences, Menlo Park, CA, USA, respectively. Reactions were carried out according to the manufacturer´s instructions. DNAs from phages were combined equimolar. SMRTbell template was exonuclease treated for removal of incompletely formed reaction products. Conditions for annealing of sequencing primers and binding of polymerase to purified SMRTbell template were assessed with the Calculator in RS Remote (Pacific Biosciences, Menlo Park, CA, USA). SMRT sequencing was carried out on the PacBio *RSII* (Pacific Biosciences, Menlo Park, CA, USA) taking one 180-minutes movie.

#### Demultiplexing, genome assembly and annotation

Data from one SMRT Cell was demultiplexed according to barcodes F1 to F3 using the “RS_Subreads.1” protocol included in SMRTPortal version 2.2.0. Hereby, the “barcoding” option was activated and “symmetric” barcoding was selected in the barcode option pulldown menu. A FASTA-file containing all barcodes was uploaded prior analysis to the “Reference” section of SMRTPortal and selected within the protocol. Output of demultiplexing workflow (barcoded-fastqs.tgz) was used to create whitelists of polymerase reads for each barcode (compare https://github.com/PacificBiosciences/Bioinformatics-Training/wiki/HGAP-Whitelisting-Tutorial). Hereby, a bash script named “Barcode_HGAP.sh “assisted in creating the necessary folder structure, generating the whitelist.txt files as well as the settings.xml file for each subsequent genome assembly. Whitelisted SMRT sequencing data from each phage was assembled independently using the “RS_HGAP_Assembly.3”protocol in SMRTPipe with minimum subread lengths of 1 kbp and an estimated genome size of 50 kbp with exception of phage vB_VmeM-32 (200 kbp). Each phage assembly revealed the fully resolved chromosomes as one contig. The assemblies where either linearized due to recognition of distinct start and end points in the phage assemblies or circularized removing artificial redundancies at the ends of the contigs. Validity of the assemblies was checked using SMRTView and IGV [56]. Finally, the genomes were annotated using Prokka 1.8 [57] with subsequent manual curation in Artemis [58].

Two criteria were used to define potential protein coding genes; they had to contain greater than 25 codons and employ ATG, GTG or TTG as initiation codons. Genome size, G+C % content, coding density, total number of genes and additional elements such as inspection of the sequence to search start and termination codons was determined using ARTEMIS tool for sequence visualization [59]. The intergenic genome regions of the phage were searched for transcriptional regulation elements. A search for tRNA genes was done with the tRNAscan-SE program v1.2.1 [60] and ARAGORN v1.2.36 [61]. Homology assignments were based on amino acid sequence alignment searches (BlastP) and were accepted only if the statistical significance of the sequence similarities (E value) was less than 1x10^-5^, the percentage query cover was ≥60% and the percentage identity between the aligned sequences was ≥35%.

#### Termini phylogenetic analysis

Sequences for termini phylogenetic analysis were chosen by large terminase gene products of BlastP. The sequences were aligned with other phage sequences with known DNA packaging strategies from a reduced set used by Fouts et al [62] using the program ClustalW [63] with default parameters in MEGA v.7 (Pairwise alignment: gap opening penalty = 10, gap extension penalty = 0.1. Multiple alignment: gap opening penalty = 10, gap extension penalty = 0.2. Protein weight matrix = Gonnet. Delay divergent cutoff = 30%) [64]. Phylogenetic tree was inferred using the Maximum—Likelihood method [65] based on the Poisson correction model [66]. Bootstrapping was set to 1000 replicates and the tree rooted.

## Results

### Isolation and characterization of bacterial host strains

Nine bacterial isolates from Lake Elmenteita were found to be susceptible to phages. They all, apart from *Vibrio metschnikovii*, were Gram-positive, grew well on alkaline nutrient (DSMZ medium 31), basal media for alkaliphilic micro-organisms; Horikoshi-1 (DSMZ medium 1081) and LB (DSMZ medium 381) media, over a temperature range of 25–45°C (optimum, 30–35°C) and pH range of 7.0–12.0 (optimum, pH 10.0–12.0). The comparative analysis of partial (approximately 900 bp) 16S rRNA gene sequences revealed that they all, apart from *Vibrio metschnikovii*, belong to the order *Bacillales*. The level of similarity between the isolates and their closest known relatives was between 98–100%. This was supported by MALDI-TOF protein spectra analysis. The bacteria showed different morphologies as indicated in Table 1.

**Table 1:** Summary of bacterial hosts’ characteristics. Hosts and their characteristic morphologies as observed under phase contrast microscope (×400).

	Host reference No.	Name	Morphology
1	HS32	*Vibrio metschnikovii*	vibrio
2	HS61	*Bacillus pseudofirmus*	short rods
3	HS123	*Exiguobacterium aurantiacum*	cocci
4	HS125	*Bacillus bogoriensis*	rods
5	HS126	*Bacillus horikoshii*	long rods
6	HS132	*Exiguobacterium alkaliphilum*	cocci
7	HS136	*Bacillus cohnii*	short rods
8	HS140	*Bacillus pseudalcaliphilus*	long-rods
9	HS171	*Bacillus halmapulus*	long rods

A summary of selected physiological properties to further characterize the isolates, as indicated by API 20NE and API ZYM (bioMérieux) identification systems are presented in S1 Table.

### Isolation and characterization of bacteriophages

**Fig 1:**
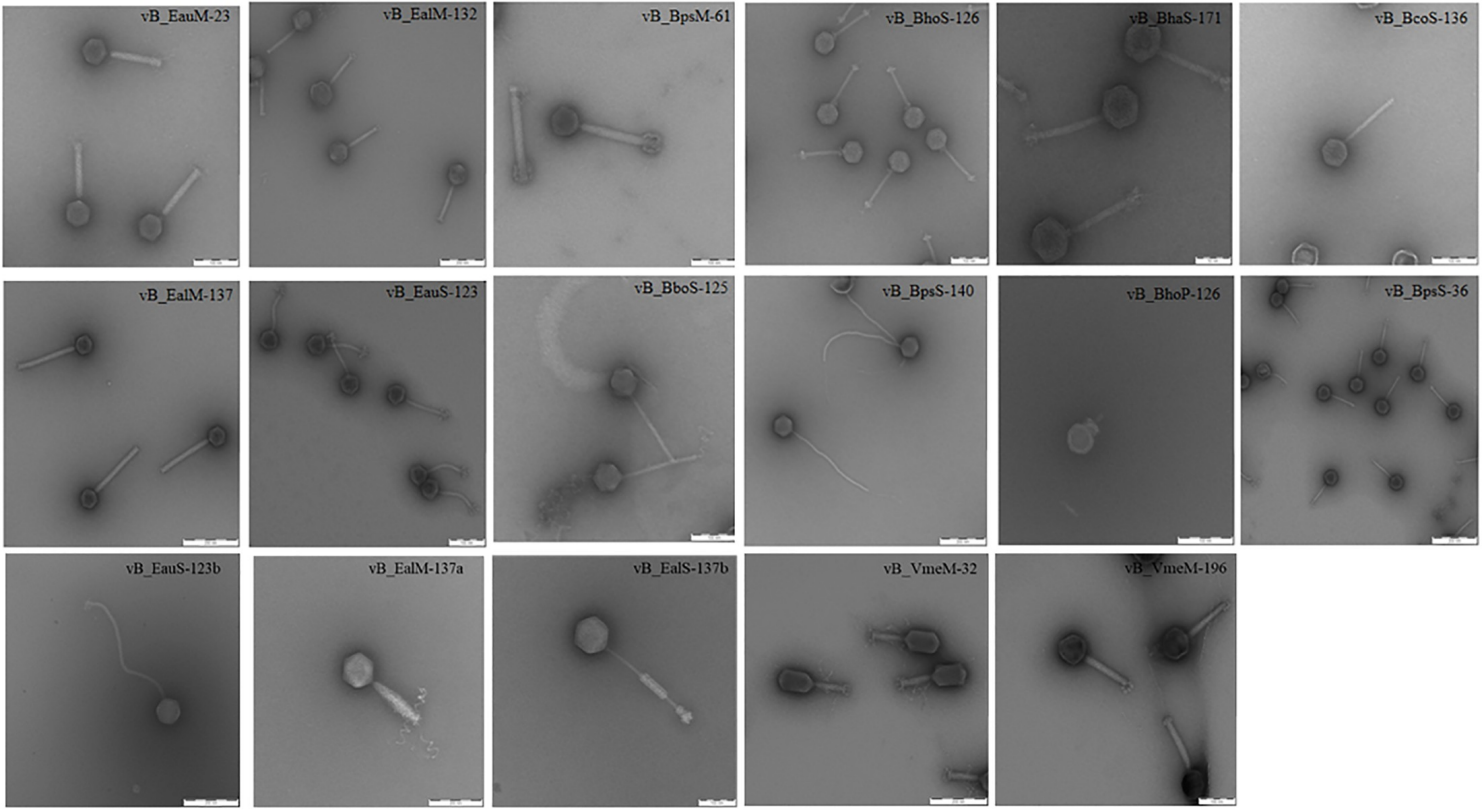
Transmission electron micrographs. Micrographs of bacteriophages from Lake Elmenteita showing different morphotypes. CsCl-purified bacteriophage preparations were negatively stained with 2% (w/v) aqueous uranyl acetate (pH 5.0). Samples were examined in a TEM 910 transmission electron microscope (Carl Zeiss, Oberkochen) at an acceleration voltage of 80 kV.

A total of 17 seemingly morphologically different phages were isolated following enrichment of sediment. Transmission electron microscope revealed tailed forms of bacteriophages with variety of structural features, to be present in this lake. According to Ackermann’s classification [67], they were all tailed phages similar to those belonging to order *Caudovirales* and consists of three families; Myoviridae, Siphoviridae and Podoviridae (Fig 1).

Among them, nine (vB_BpsS-36, vB_EauS-123a, vB_EauS-123b, vB_BboS-125, vB_BhoS-126, vB_BcoS-136, vB_BpsS-140, vB_BhaS-171 and vB_EalS-137b) were siphoviruses, seven (vB_EauM-23, vB_VmeM-32, vB_BpsM-61, vB_EalM-132, vB_EalM-137, vB_EalM-137a and vB_VmeM-196) myoviruses and one (vB_BhoP-126) podovirus. The capsid diameters ranged between 47–130 nm and tail lengths measured from the bottom of the neck to the base plate ranged between 37–546 nm. The bacteriophages were further named according to the recommendations outlined by Kropinski et al [68]. Results are summarized in Table 2.

**Table 2:** Bacteriophage morphology and naming. Structural characterization, classification and naming of phages isolated from Lake Elmenteita.

Host		Family	Name	Phage size (nm)		
				Head diameter	Tail length	Total size
1	*Bacillus pseudofirmus*	myovirus	vB_BpsM-61	66	192	258
2	*Exiguobacterium aurantiacum*	myovirus	vB_EauM-23	60	125	185
3	*Exiguobacterium aurantiacum*	siphovirus	vB_EauS-123	49	138	187
4	*Exiguobacterium aurantiacum*	siphovirus	*vB_EauS-123b	-	-	-
5	*Bacillus bogoriensis*	siphovirus	vB_BboS-125	61	179	240
6	*Bacillus horikoshii*	siphovirus	vB_BhoS-126	57	119	177
7	*Bacillus horikoshii*	podovirus	vB_BhoP-126	47	37	85
8	*Exiguobacterium alkaliphilum*	myovirus	vB_EalM-132	85	161+160	406
9	*Bacillus cohnii*	siphovirus	vB_BcoS-136	59	145	204
10	*Exiguobacterium alkaliphilum*	myovirus	*vB_EalM-137a	-	-	-
11	*Exiguobacterium alkaliphilum*	siphovirus	*vB_EalS-137b	-	-	-
12	*Exiguobacterium alkaliphilum*	myovirus	vB_EalM-137	62	214	276
13	*Bacillus pseudalcaliphilus*	siphovirus	vB_BpsS-140	83	546+179	809
14	*Bacillus halmapulus*	siphovirus	vB_BhaS-171	58	117	175
15	*Bacillus pseudalcaliphilus*	siphovirus	vB_BpsS-36	57	110	167
16	*Vibrio metschnikovii*	myovirus	vB_VmeM-32	130	109	239
17	*Vibrio metschnikovii*	myovirus	vB_VmeM-196	77	159	236

*The phage dimension was not determined as the culture was not clonal

### Thermal and pH stability tests

**Fig 2:**
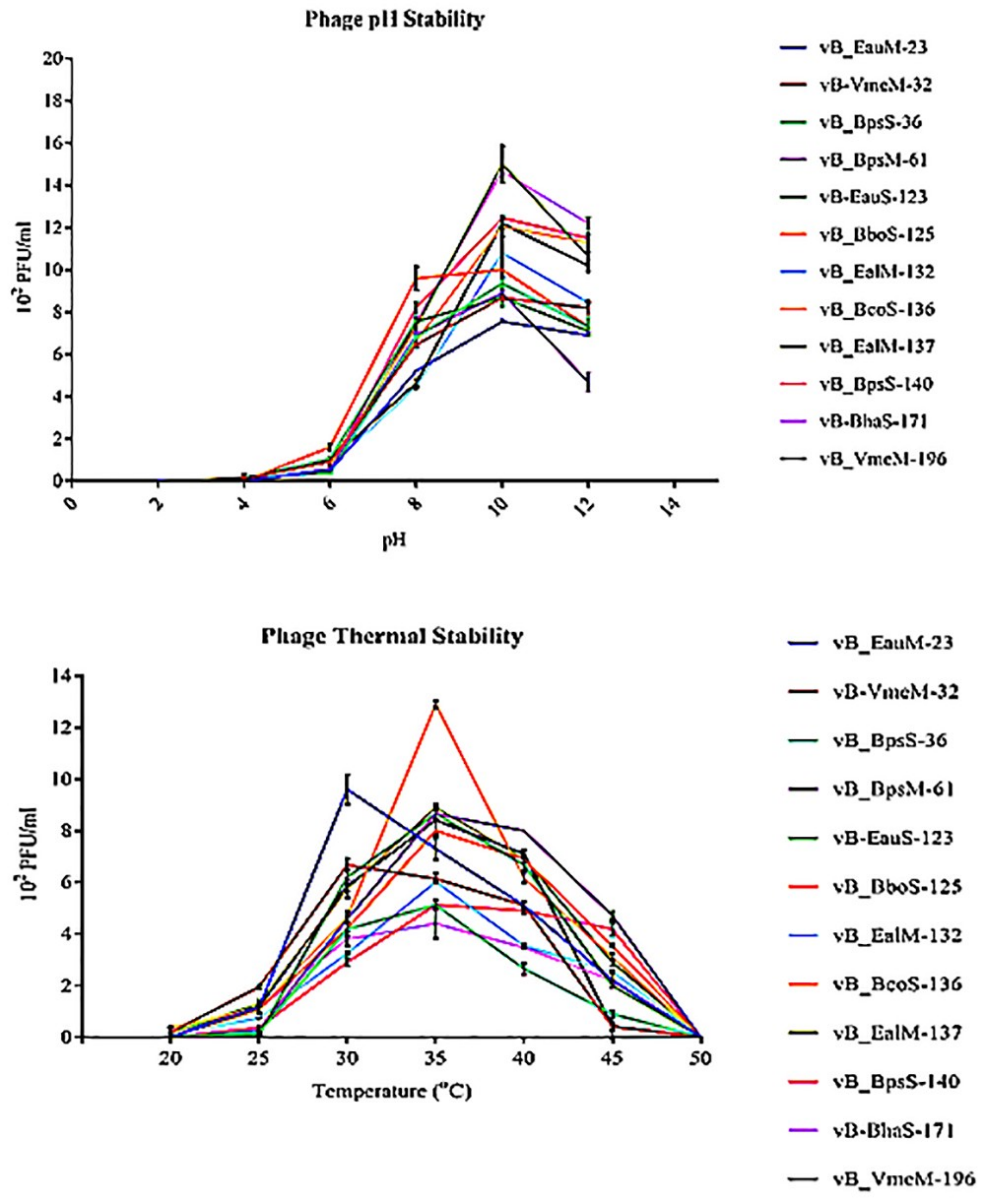
Thermal and pH stability experiments. Infection capability of the phages was highest at 30–35°C and more infective in alkali condition of between pH 10–12.

After 6 h of incubation under different thermal conditions, at 20°C generally the phages had lost infectivity as no plaques formed. Plaque forming units however increased exponentially from 25°C with maximum at 35°C. The infectivity of the phages was highest at 30–35°C, but was lost with increasing temperature with non at 50°C as they had lost their infection capability. After 6 h of incubation under different pH conditions, at pH 2 and 4 no plaques were observed as the infectivity had been hindered by the low pH. Plaques formed from pH 7 and increased with increasing pH values with maximum at pH 10. Infection capability of the phages was highest between pH 10–12 (Fig 2).

Host range analysis revealed that the bacteriophages exhibited high specificity for their host bacteria and did not infect other bacteria strain tested (S2 Table).

### Structural protein profiles

**Fig 3:**
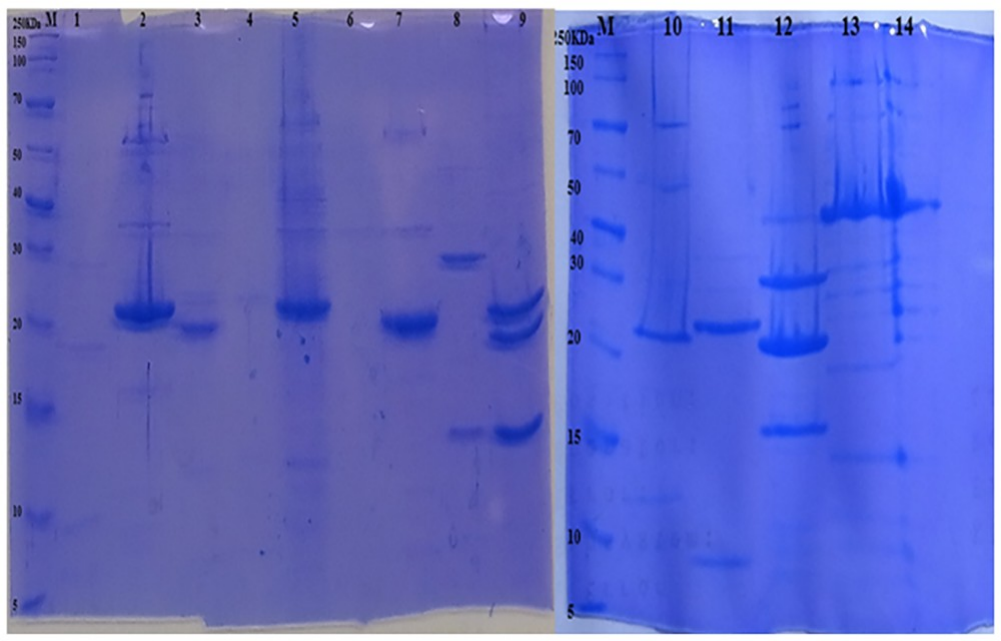
SDS-polyacrylamide gel electrophoretic profiles of phage structural proteins. (M) broad range Page Ruler protein molecular weight marker (Thermoscientific), (1) vB_BpsM-61, (2) vB_EauM-23, (3) vB_EauS-123, (4) vB_BboS-125, (5) vB_BhoP-126, (6) vB_BhoP-126, (7) vB_EalM-132, (8) vB_BcoS-136, (9) vB_EalM-137, (10) vB_BpsS-140, (11) vB_BhaS-171, (12) vB_BpsS-36, (13) vB_VmeM-32, (14) vB_VmeM-196, Numbers to the left indicate band size in kDa.

The molecular weights of the structural polypeptides ranged from 10 to 100 kDa. While the minor bands varied in position, most phages had the major band at 20 kDa (Fig 3).

### Genome size estimation

**Fig 4:**
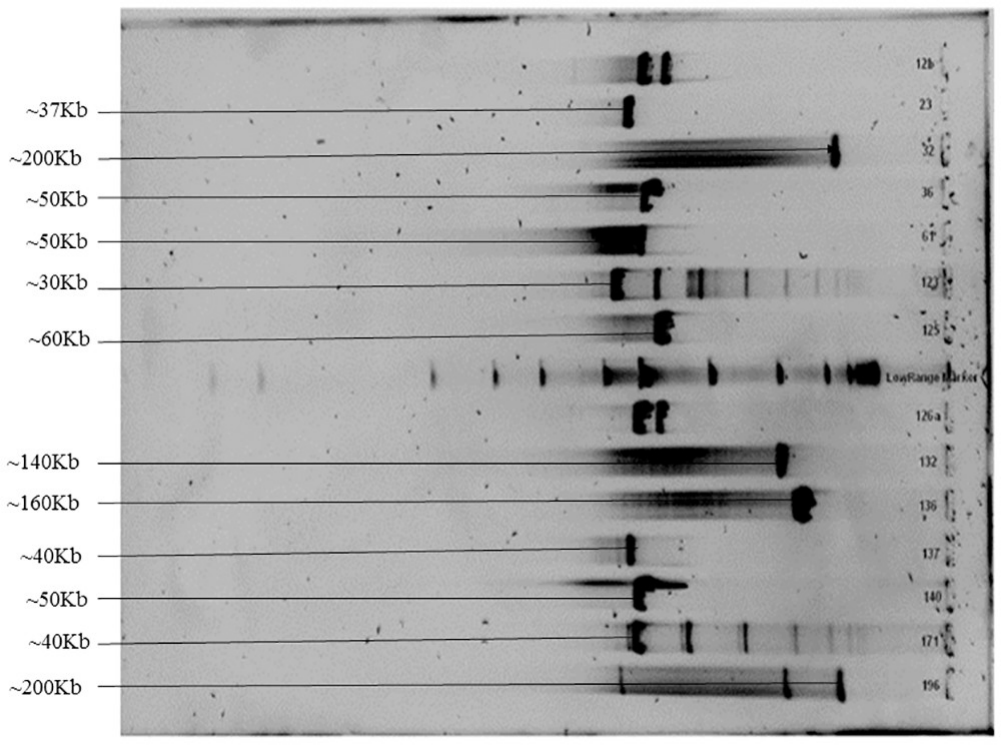
Pulsed-field gel of the 13 phage genomes. Lane, (1) vB_EauM-23, (2) vB_VmeM-32, (3) vB_BpsS-36, (4) vB_BpsM-61, (5) vB_EauS-123, (6) vB_BboS-125, (7) vB_BhoP-126, (8) vB_EalM-132, (9) vB_BcoS-136, (10) vB_EalM-137, (11) vB_BpsS-140, (12) vB_BhaS-171, (13) vB_VmeM-196, (M) Low range PFGE DNA marker in Kb (Biolabs, England).

The genome sizes of all the 12 phages ranged between ~30 to 200 kb. Bacteriophages vB_VmeM-32, vB_EalM-132, vB_BcoS-136 and vB_VmeM-196 had the largest genomes of this study ranging between ~140 to 200 kb. The rest of the phages had genome sizes ranging between ~30 to 60 kb (Fig 4).

### Restriction digestion patterns

The patterns of restriction digest profiles for each phage were different (S1 Fig). Endonuclease *Eco*RI was able to digest all genomes. *Dra*I also digested all the genomes but vB_BpsS-140. Most phages showed insensitivity to restriction endonucleases *Pst*I (all but vB_EauM-23, vB_BboS-125 and vB_BpsS-36) and *BamH1* (all but vB_BpsM-61) (S3 Table). Restriction digests further confirm that the phages are double stranded DNA viruses.

### Genome characteristics and annotation

The genomes of phages vB_VmeM-32, vB_EauS-123 and vB_BhaS-171 have genome sizes of 199,912 bp, 30,925 bp, and 38,975 bp, respectively. A total of 260 open reading frames (ORFs) were predicted for phage vB_VmeM-32 while 56 ORFs were predicted for vB_EauS-123 and 67 ORFs for vB_BhaS-171. Phages vB_VmeM-32 and vB_EauS-123 encoded 6 transcriptional terminators each and vB_BhaS-171 encoded 5 transcriptional terminators. 3 tRNA genes (Met_cat_, Arg_tct_ and Asn_gtt_) were detected in the genome of phage vB-VmeM-32 clustered at region 27879–28124 bp, while vB_EauS-123 and vB_BhaS-171 did not encode any tRNA gene. See summary in Table 3.

**Table 3:** General features of genomes vB_VmeM-32, vB_EauS-123 and vB_BhaS-171. A summary of genome characteristics.

	Phage	Genome size (bp)	G +C % content	Coding %	CDS	tRNAs	Transcriptional Terminators	Start Codon		
								ATG	GTG	TTG
1	vB-VmeM-32	199, 912	36.25	91.2	260	3	48	246	3	11
2	vB_EauS-123	30, 925	47.73	91.5	56	-	6	52	2	2
3	vB_BhaS-171	38, 975	40.82	91.6	67	-	5	50	8	9

**Fig 5:**
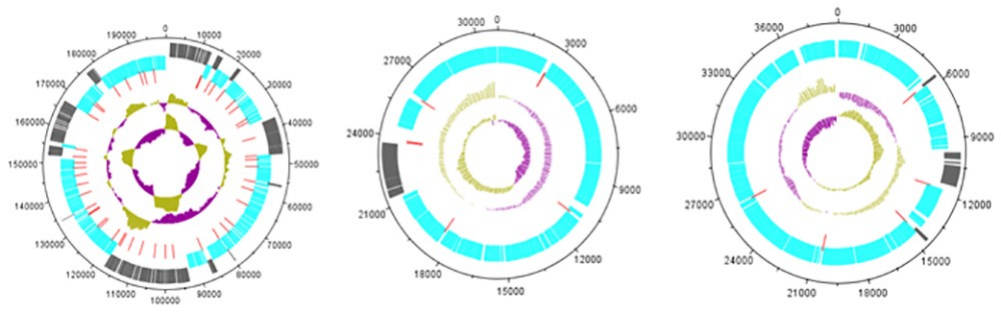
Genome maps of bacteriophages vB-VmeM-32, vB_EauS-123 and vB_BhaS-171 respectively, drawn to scale. First track show forward transcribed ORFs and second tracks show reverse transcribed ORFs respectively. Third track shows terminators (red). Moving inward, the track show the %GC content (purple = low %GC) and innermost of the genome map GC skew ([G-C]/[G+C]).

Most of the ORFs of phages vB_EauS-123 and vB_BhaS-171 are located on the reverse (minus) strand while vB-VmeM-32 has all its genes transcribed on the forward (plus) strand (Fig 5).

Based on sequence similarity (E value < 10^−5^), 135 out of 260 (52%), 40 out of 66 (61%) and 24 out of 55 (44%) of the protein-coding genes for phages vB-VmeM-32, vB_EauS-123 and vB_BhaS-171, respectively, share significant sequence similarity to known protein sequences contained in the GenBank non-redundant protein database. Genome wide comparison of the phages with other genomes in the non-redundant NCBI database showed no significant sequence similarity hence novel. Further analysis of vB_VmeM-32 genome revealed genes with suggested functions like putative N-acetylmuramoyl-L-alanine amidase (VmeM-32_00065) for host cell lysis and a putative DNA polymerase (VmeM-32_00094) for replication. Gene for a putative helicase (VmeM-32_00016), exonuclease protein (VmeM-32_00081) and endonuclease protein (VmeM-32_00138) were also identified. We identified structural genes with conserved domains in phage vB_EauS-123 that showed no similarities to other phages (EauS-123_00048, EauS-123_00051). Further analysis of this genome revealed few more genes for proteins with suggested functions like a putative N-acetylmuramoyl-L-alanine amidase (EauS-123_00053) for host cell lysis, a putative recombinase (EauS-123_00055), a putative phage regulatory protein (EauS-123_00008), a putative Holliday junction resolvase (EauS-123_00012), a putative dUTPase (EauS-123_00020) and two proteins for replication, containing a DnaC (EauS-123_00004) and DnaD domain (EauS-123_00003) respectively. The rest of genome did not show any similarity to any other genes with known functions so far. Most of the few similarities vB_BhaS-171 shared with other phages were assigned to temperate phage 11143 that was induced from *Bacillus cereus* strain NCTC11143 [69]. These included genes of the cluster for DNA packaging and head morphogenesis (BhaS-171_00005 and BhaS-171_00012), e.g. genes for two terminase subunits and a portal protein, and a gene for a putative helicase (BhaS-171_00053). Generally, vB_BhaS-171 had a typical gene cluster for head and tail proteins, though most of those genes were annotated based on conserved domains at amino acid level and not based on similarities to other known viruses. Downstream the lysis cluster, we also identified genes for an FtsK/SpoIIIE-like protein and a putative replication/relaxation protein similar to vB_BpsS-36. The replication cluster revealed some genes similar to deep-sea thermophilic bacteriophage GVE2 [69][48], e.g. for a helicase (BhaS-171_00053), a Ssb protein (BhaS-171_00061) and an endonuclease (BhaS-171_00057). Additionally, this phage harbored a gene with a conserved domain for an NTP-PPase and a cytosine-C5 specific DNA methylase. A comprehensive list of protein coding genes carried by the phages along with the corresponding positions, sizes, and sequence homologies are presented in S4, S5 and S6 Tables.

**Fig 6:**
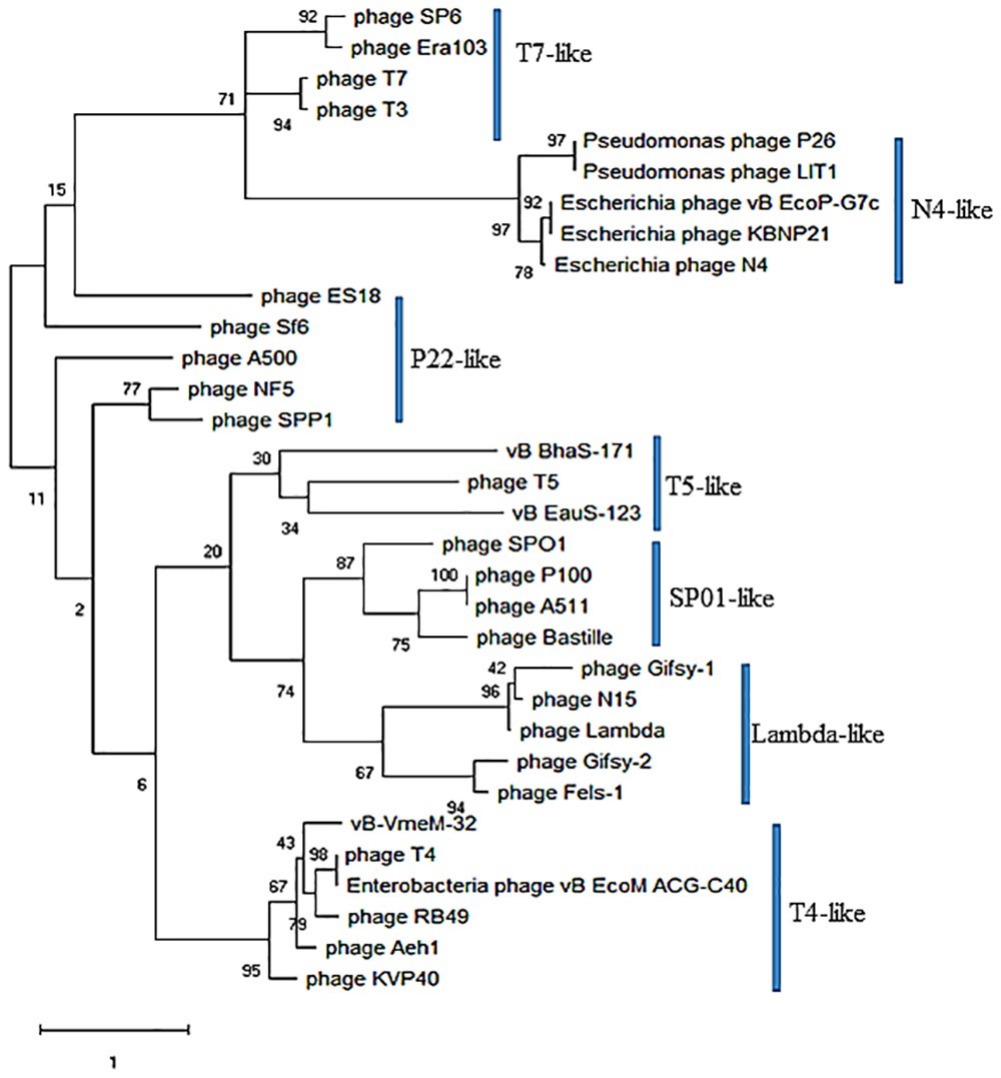
Phylogenetic analysis of large terminase subunits compared to phages with known DNA packaging strategies. The maximum—Likelihood tree was inferred based on ClustalW alignment of large terminase subunits amino acid sequences. The tree was rooted via midpoint rooting. The numbers at the nodes represent bootstrap values based on 1,000 resamplings.

A phylogenetic tree for large terminase subunit generated using Maximum—Likelihood method revealed that phage vB_VmeM-32 cluster together with T4-like phages with a low bootstrap value of 43%, while Bacillus phages vB_BhaS-171 and vB_EauS-123 clustered with T5-like phages with low bootstrap values of 34% and 30% respectively (Fig 6).

## Discussion

Nine bacterial host strains obtained in this study from the haloalkaline lake Elmenteita, showed physiological characteristics similar to previously isolated bacteria from this lake [70] which include growth in alkaliphilic conditions and temperatures above 30°C, with order *Bacillales* being the most abundant and easily isolated bacteria [33][34].

Besides genomics, the most important criterion for phage taxonomy is ultrastructure [67]. The phenotypic diversity of the 17 bacteriophages was examined by electron microscopy. The phages were identified using morphological criteria outlined by the International Committee of Taxonomy of Viruses and the species concept of Ackermann et al [67]. All the bacteriophages belong to the order Caudovirales characterized by tailed phages. The order has three common virus types; myoviruses, siphoviruses and podoviruses. Siphovirus and myovirus phages were prevalent morphotypes compared to podovirus. Most of the phages have morphological features that have been described previously from a marine environment by Sime-ngando et al [71]. Few showed unique structures that have not been reported previously for haloalkaliphilic viruses. The unique structural features, different phage dimensions and plaque morphology all indicate diversity within the various families. The stability of phages under different thermal and pH conditions was investigated based on infectivity of phages after treatment. Infection capability of the phages was highest at 30–35°C. Phages were more infective in alkali than acidic environment. Optimal temperature and pH for bacteriophage growth and plaque formation was similar to that of bacterial host strains.

The bacteriophage exhibited high specificity for their hosts. This characteristic has been previously reported for marine phages by Børsheim [72]. Because of their specificity, the phages can therefore be applied to map the distribution of bacteria. Phage mapping is a very sensitive tool for tracing specific groups of bacteria, compared to using taxonomy of bacteria [72].

Protein profiles assessment exhibited variations. The molecular weights of the structural polypeptides as indicated by SDS-PAGE, ranged from 10 to 100 kDa. While the minor bands varied in position, most phages had the major band at 20 kDa, which might represent the major capsid protein. The minor bands were varied and might be responsible for host-specificity or the characteristics specific to a particular phage [73]. Resistance to restriction enzymes as indicated by the phages of this study to endonucleases *Pst*l and *Bam*H1 is common and has been reported previously [74][75]. Several explanations have been proposed for this anti-restriction mechanisms. Among these explanations is elimination of restriction sites as an evolutionary response of phages to pressures from their host restriction enzymes [76], integration of unusual bases in the viral DNA such as hydroxymethyl uracil or hydroxymethyl cytosine that make DNA somewhat refractory to endonuclease cleavage. Alternatively, phage genomes may encode methyltransferases that modify specific nucleotides within the recognition site of one or more of the restriction endonucleases [77][78].

28% of the vB_VmeM-32 genes would result in proteins of less than 100 amino acid residues. 58.5% of putative vB_VmeM-32 genes resulted in BLASTp hits with identities to various biological groups (Phage and Bacteria) but few lacked any database matches (Hypothetical). The phage genes were further subdivided on the basis of their best blastp hit (BLASTp values E >10^−5^). A high degree of similarity in most protein coding genes was with a Schizo T-even *Aeromonas* phage Aeh1, an *Aeromonas hydrophila* phage isolated from a sewage treatment plant in Wisconsin [79]. SchizoT-evens phages comprise the subgroup of T4-types that have diverged significantly from the T-evens and infect host distant from *E*. *coli* e.g., *Aeromonas* and *Vibrio* [80]. Genome sequencing and assembly of Phage vB_BhaS-171 and phage vB_EauS-123 and comparison with other phage genomes via BLASTN showed they share only few similarities with other phages.

The large terminase sub-unit is considered the most universally conserved gene sequence in phages hence used to construct phylogeny to decipher evolutionary relationships among phages belonging to different families [79][81]. Casjens and Gilcrease [82] have also shown the phylogeny of large terminase sub-unit proteins is correlated with the virus DNA packaging strategy. Since the sequence of Vibrio phage vB-VmeM-32 clustered phylogenetically with the T4-like phages which are known to package DNA by a headful packaging mechanism, we therefore conclude that phage vB-VmeM-32 also package DNA by the same headful packaging mechanism. Phage vB_VmeM-32 can therefore be classified as a new member of the T4-like phages, subgroup Schizo T-evens, and infecting bacteria of the genus *Vibrio*. vB_EauS-123 and vB_BhaS-171 clustered with T5-like phages which show long exact direct repeat ends mechanism of DNA packaging.

## Conclusion

The effective use of bacteriophage in all applications must be preceded by detailed understanding of the bacteriophages themselves and analysis of their physiologic characteristics. Isolation, characterization and comparative analysis of phages were the main accomplishments of this study, as an outcome the phages turned out to be different in identity. The taxonomic grouping based upon ultrastructural characteristics, structural proteins, restriction endonuclease patterns and genome size analysis is therefore an effective approach to the classification of the phages. Although we investigated only a small part of the viral community, we established that there is great morphological and genetic variation in the bacteriophages, which leads to high levels of species and strain diversity. Molecular studies of the phages based on GC-ratios, and DNA-DNA similarity between the phages is necessary to confirm the taxonomic status of the groups and provide more information into interaction of phages and hosts. Genome sequencing and computational analysis of the three phages revealed basic and important information about the DNA structure, genome organization and layout and phage relatedness. Further investigations of phage ecology are also recommended in order to gain a more complete understanding of microbial interactions in Lake Elmenteita.

## Nucleotide sequence accession numbers

The bacteriophages were accessed to the German Collection of Microorganisms and Cell Cultures (DSMZ) under the following Accession numbers: vB_EauM-23 (DSM 29710), vB_VmeM-32 (DSM 29703), vB_BpsS-36 (DSM 29701), vB_BpsM-61 (DSM 29705), vB_EauS-123 (DSM 29709), vB_BboS-125 (DSM 29706), vB_BhoS-126a (DSM 29707), vB_BhoP-126b (DSM 29708), vB_BcoS-136 (DSM 29699), vB_BpsS-140 (DSM 29700), vB_BhaS-171 (DSM 29702), vB_PmeM-196 (DSM 29704) and the genome sequences deposited at NCBI GenBank under the accession numbers vB_VmeM-32 (KU160494), vB_EauS-123 (KU160495) and vB_BhaS-171 (KU160496).

## Supporting information

S1 Fig Restriction profiles. Restriction profiles of the phages after digestion of DNA with restriction enzymes, overnight at 37°C and electrophoresed on 1% agarose gel. Different restriction enzymes were used which cut wherever the recognition sequence was present. (A) *Dra*I, (B) *Kpn*I, (C) *Pst*I (D) *Hind*III (E) *Eco*RI and (F) BamH1 all from Fermentas. Lane (1) vB_BpsM-61, (2) vB_EauM-23, (3) vB_EauS-123, (4) vB_BboS-125, (5) vB_BhoP-126, (6) vB_BhoP-126, (7) vB_EalM-132, (8) vB_BcoS-136, (9) vB_EalM-137, (10) vB_BpsS-140, (11) vB_BhaS-171, (12) vB_BpsS-36, (13) vB_VmeM-32, (14) vB_VmeM-196, (M) 1kb DNA marker (Metabione). Numbers to the right indicate band size in kb.
(TIF)

S1 Table Physiological properties. Selected phenotypic characteristics of host bacteria as indicated by API identification system.
(DOCX)

S2 Table Host range analysis of bacteriophages. Evaluation of the lytic spectrum of the phages against bacterial strains isolated in this study.
(DOCX)

S3 Table Grouping of restriction endonucleases by cutting pattern. Non cutters produced only one high molecular weight band by gel electrophoresis. Poor cutters produced few bands, good cutters produced five or more bands and complete cutters caused complete digestion of DNA.
(DOCX)

S4 Table Overview of bacteriophage vB_VmeM-32 ORFS and summary of homology searches. ORFs are arranged according to their position (Start-End) in the genome. Significant database matches are given in the column marked Putative homolog. Tools used to search for similarity are blastn (nucleotide Blast search) or blastp (protein Blast search). Scores and E-values obtained in the Blast searches are given in the last three columns. Homology assignments were accepted only if the statistical significance of the sequence similarities (E value) was less than 1x10^-5^, the percentage query cover was ≥60% and the percentage identity between the aligned sequences was ≥35%.
(DOCX)

S5 Table Overview of bacteriophage vB_EauS-123 ORFS and summary of homology searches. ORFs are arranged according to their position (Start-End) in the genome. Significant database matches are given in the column marked Putative homolog. Tools used to search for similarity are blastn (nucleotide Blast search) or blastp (protein Blast search). Scores and E-values obtained in the Blast searches are given in the last three columns. Homology assignments were accepted only if the statistical significance of the sequence similarities (E value) was less than 1x10^-5^, the percentage query cover was ≥60% and the percentage identity between the aligned sequences was ≥35%.
(DOCX)

S6 Table Overview of bacteriophage vB_BhaS-171 ORFS and summary of homology searches. ORFs are arranged according to their position (Start-End) in the genome. Significant database matches are given in the column marked Putative homolog. Tools used to search for similarity are blastn (nucleotide Blast search) or blastp (protein Blast search). Scores and E-values obtained in the Blast searches are given in the last three columns. Homology assignments were accepted only if the statistical significance of the sequence similarities (E value) was less than 1x10^-5^, the percentage query cover was ≥60% and the percentage identity between the aligned sequences was ≥35%.
(DOCX)
