# Supplementary figures and images for: Isolation, characterization and analysis of bacteriophages from the haloalkaline lake Elmenteita, Kenya
Source: PLoS One. 2019 Apr 25;14(4):e0215734. doi: 10.1371/journal.pone.0215734 (PMC6483233; doi:10.1371/journal.pone.0215734)

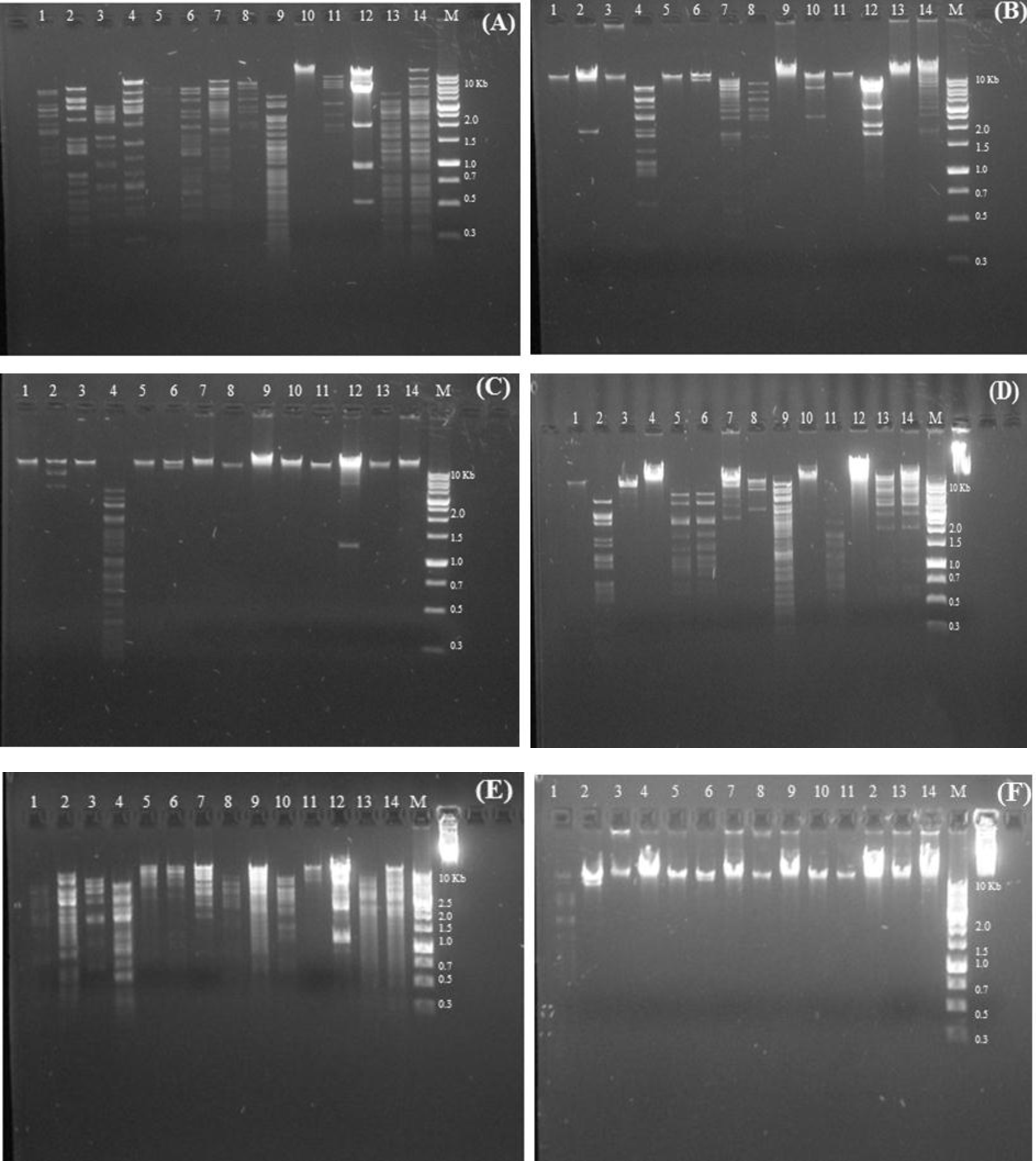

Supplement: S1 Fig — Restriction profiles of the phages after digestion of DNA with restriction enzymes, overnight at 37°C and electrophoresed on 1% agarose gel. Different restriction enzymes were used which cut wherever the recognition sequence was present. (A) DraI, (B) KpnI, (C) PstI (D) HindIII (E) EcoRI and (F) BamH1 all from Fermentas. Lane (1) vB_BpsM-61, (2) vB_EauM-23, (3) vB_EauS-123, (4) vB_BboS-125, (5) vB_BhoP-126, (6) vB_BhoP-126, (7) vB_EalM-132, (8) vB_BcoS-136, (9) vB_EalM-137, (10) vB_BpsS-140, (11) vB_BhaS-171, (12) vB_BpsS-36, (13) vB_VmeM-32, (14) vB_VmeM-196, (M) 1kb DNA marker (Metabione). Numbers to the right indicate band size in kb. (TIF) [file pone.0215734.s001.tif]
