# Supplementary material for: Isolation, characterization and analysis of bacteriophages from the haloalkaline lake Elmenteita, Kenya
Source: PLoS One. 2019 Apr 25;14(4):e0215734. doi: 10.1371/journal.pone.0215734 (PMC6483233; doi:10.1371/journal.pone.0215734)
Supplement: S1 Table — Selected phenotypic characteristics of host bacteria as indicated by API identification system. (DOCX) [file pone.0215734.s002.docx]

**Supplementary Table 1: Physiological properties.** Selected phenotypic characteristics of host bacteria as indicated by API identification system.

| Substrate | **Test organisms** | | | | | | | | |
| --- | --- | --- | --- | --- | --- | --- | --- | --- | --- |
|  | **HS32** | **HS61** | **HS123** | **HS125** | **HS126** | **HS132** | **HS136** | **HS140** | **HS171** |
| **API 20NE** | | | | | | | | | |
| Indole production | + | ++ | - | + | ++ | + | ++ | + | + |
| Glucose fermentation | + | - | + | - | + | + | - | - | + |
| Arginine dihydrolase | + | - | + | + | + | ++ | - | + | + |
| Urease production | + | - | + | + | - | + | - | + | ++ |
| Esculin hydrolysis | ++ | ++ | ++ | ++ | ++ | + | + | ++ | ++ |
| Gelatin hydrolysis | ++ | + | - | + | + | + | - | + | ++ |
| **API ZYM** | | | | | | | | | |
| Alkaline phosphatase | + | + | ++ | ++ | ++ | ++ | ++ | ++ | ++ |
| Esterase C4 | + | + | + | ++ | ++ | + | + | + | + |
| Lipase C8 | - | + | ++ | ++ | ++ | + | + | + | + |
| Leucine aminopeptidase | + | ++ | ++ | ++ | ++ | ++ | ++ | ++ | ++ |
| Valine aminopeptidase | + | ++ | - | ++ | ++ | + | + | + | ++ |
| Cystine aminopeptidase | - | ++ | - | + | ++ | + | - | + | - |
| Trypsin | - | + | - | ++ | ++ | - | + | + | ++ |
| α -galactosidase | ++ | - | + | ++ | - | - | ++ | - | - |
| β -galactosidase | ++ | - | ++ | ++ | + | ++ | - | - | - |
| α-glucosidase | + | ++ | ++ | ++ | + | ++ | ++ | ++ | - |
| β -glucosidase | ++ | ++ | ++ | ++ | ++ | ++ | + | - | - |

Key: -, no reaction; +, good; ++, excellent.
