# Supplementary material for: Isolation, characterization and analysis of bacteriophages from the haloalkaline lake Elmenteita, Kenya
Source: PLoS One. 2019 Apr 25;14(4):e0215734. doi: 10.1371/journal.pone.0215734 (PMC6483233; doi:10.1371/journal.pone.0215734)
Supplement: S2 Table — Evaluation of the lytic spectrum of the phages against bacterial strains isolated in this study. (DOCX) [file pone.0215734.s003.docx]

**Supplementary Table 2: Host range analysis of bacteriophages**. Evaluation of the lytic spectrum of the phages against bacterial strains isolated in this study.

|  |  | **Bacteriophages** | | | | | | | | | | | |
| --- | --- | --- | --- | --- | --- | --- | --- | --- | --- | --- | --- | --- | --- |
|  | **Hosts** | vB_EauM-23 | vB_VmeM-32 | vB_BpsS-36 | vB_BpsM-61 | vB_EauS-123 | vB_BboS-125 | vB_EalM-132 | vB_BcoS-136 | vB_EalM-137 | vB_BpsS-140 | vB_BhaS-171 | vB_PmeM-196 |
| 1 | *Vibrio metschnikovii* | **-** | **+** | **-** | **-** | **-** | **-** | **-** | **-** | **-** | **-** | **-** | **+** |
| 2 | *Bacillus pseudofirmus* | **-** | **-** | **-** | **+** | **-** | **-** | **-** | **-** | **-** | **-** | **-** | **-** |
| 3 | *Exiguobacterium aurantiacum* | **+** | **-** | **-** | **-** | **+** | **-** | **-** | **-** | **-** | **-** | **-** | **-** |
| 4 | *Bacillus bogoriensis* | **-** | **-** | **-** | **-** | **-** | **+** | **-** | **-** | **-** | **-** | **-** | **-** |
| 5 | *Bacillus horikoshii* | **-** | **-** | **-** | **-** | **-** | **-** | **-** | **-** | **-** | **-** | **-** | **-** |
| 6 | *Exiguobacterium alkaliphilum* | **-** | **-** | **-** | **-** | **-** | **-** | **+** | **-** | **+** | **-** | **-** | **-** |
| 7 | *Bacillus cohnii* | **-** | **-** | **-** | **-** | **-** | **-** | **-** | **+** | **-** | **-** | **-** | **-** |
| 8 | *Bacillus pseudalcaliphilus* | **-** | **-** | **+** | **-** | **-** | **-** | **-** | **-** | **-** | **+** | **-** | **-** |
| 9 | *Bacillus halmapulus* | **-** | **-** | **-** | **-** | **-** | **-** | **-** | **-** | **-** | **-** | **+** | **-** |

**Key:** +, susceptible -, not susceptible
