# Supplementary material for: Isolation, characterization and analysis of bacteriophages from the haloalkaline lake Elmenteita, Kenya
Source: PLoS One. 2019 Apr 25;14(4):e0215734. doi: 10.1371/journal.pone.0215734 (PMC6483233; doi:10.1371/journal.pone.0215734)
Supplement: S3 Table — Non cutters produced only one high molecular weight band by gel electrophoresis. Poor cutters produced few bands, good cutters produced five or more bands and complete cutters caused complete digestion of DNA. (DOCX) [file pone.0215734.s004.docx]

**Supplementary Table 3: Grouping of restriction endonucleases by cutting pattern.** Non cutters produced only one high molecular weight band by gel electrophoresis. Poor cutters produced few bands, good cutters produced five or more bands and complete cutters caused complete digestion of DNA.

| No. | Phage | Non cutter | Poor cutter | Good cutter | Complete cutter |
| --- | --- | --- | --- | --- | --- |
| 1 | **vB_EauM-23** | *Bam*H1 | *Kpn*l, *Pst*l |  | *Dra*l, *Hind*lll, *Eco*Rl |
| 2 | **vB_VmeM-32** | *Kpn*l, *Pst*l, *Bam*H1 |  |  | *Dra*l, *Hind*lll, *Eco*Rl |
| 3 | **vB_BpsS-36** | *Hindlll, BamH1* | Pstl | Dral, Kpnl | *EcoRl* |
| 4 | **vB_BpsM-61** | *Kpn*l, *Pst*l, *Hind*lll, | *Bam*H1 |  | *Dra*l, *Eco*Rl |
| 5 | **vB_EauS-123** | *Kpn*l, *Pst*l, *Hind*lll, *Bam*H1 |  |  | *Dra*l, *Eco*Rl |
| 6 | **vB_BboS-125** | *Hind*lll, *Bam*H1 |  |  | *Dral, Kpn*l, *Pst*l, *Eco*Rl |
| 7 | **vB_EalM-132** | *Pst*l, *Bam*H1 |  |  | *Dral, Kpn*l, *Hind*lll, *Eco*Rl |
| 8 | **vB_BcoS-136** | *Pst*l, BamH1 |  | *Hind*lll | *Dral, Kpn*l, *Eco*Rl |
| 9 | **vB_EalM-137** | *Kpn*l, *Pst*l, *Bam*H1 |  |  | *Dra*l, *Hind*lll, *Eco*Rl |
| 10 | **vB_BpsS-140** | *Dra*l, *Pst*l, *Hind*lll*, Bam*H1 | *Kpn*l |  | *Eco*Rl |
| 11 | **vB_BhaS-171** | *Kpn*l, *Pst*l*, Bam*H1 | *Eco*Rl |  | *Dra*l, *Hind*lll |
| 12 | **vB_PmeM-196** | *Pst*l, *Bam*H1 |  | *Kpn*l | *Dra*l, *Hind*lll, *Eco*Rl |
