# Supplementary material for: Isolation, characterization and analysis of bacteriophages from the haloalkaline lake Elmenteita, Kenya
Source: PLoS One. 2019 Apr 25;14(4):e0215734. doi: 10.1371/journal.pone.0215734 (PMC6483233; doi:10.1371/journal.pone.0215734)
Supplement: S4 Table — ORFs are arranged according to their position (Start-End) in the genome. Significant database matches are given in the column marked Putative homolog. Tools used to search for similarity are blastn (nucleotide Blast search) or blastp (protein Blast search). Scores and E-values obtained in the Blast searches are given in the last three columns. Homology assignments were accepted only if the statistical significance of the sequence similarities (E value) was less than 1x10^-5^, the percentage query cover was ≥60% and the percentage identity between the aligned sequences was ≥35%. (DOCX) [file pone.0215734.s005.docx]

**Supplementary Table 4:** Overview of bacteriophage vB-VmeM-32 ORFs and summary of homology searches.

| **Orf no.** | **Strand** | **Start**  **codon** | **Start End** | **aa** | **Function** | **Putative homolog** | **Accession no.** | **% Identity** | **% Query cover** | **E-value** |
| --- | --- | --- | --- | --- | --- | --- | --- | --- | --- | --- |
| 1 | + | ATG | 1102 3216 | 704 | large terminase protein | gp17 terminase DNA packaging enzyme lage subunit (Aeromonas phage Aeh1) | [NP-944105.1](http://www.ncbi.nlm.nih.gov/protein/38640149?report=genbank&log$=prottop&blast_rank=1&RID=T76AAAJJ01R) | 65 | 62 | 7e -154 |
| 2 | + | ATG | 3262 5289 | 675 | tail sheath protein | [gp18 tail sheath protein [Aeromonas phage PX29]](http://blast.ncbi.nlm.nih.gov/blast/Blast.cgi#alnHdr_593774019) | [YP-009011642.1](http://www.ncbi.nlm.nih.gov/protein/593774019?report=genbank&log$=prottop&blast_rank=2&RID=T7P1JETD01R) | 58 | 99 | 0 |
| 3 | + | ATG | 5353 6177 | 274 | hypothetical protein | - | - | - | - | - |
| 4 | + | ATG | 6498 6986 | 162 | tail tube protein | [tail tube protein [Pectobacterium bacteriophage PM2]](http://blast.ncbi.nlm.nih.gov/blast/Blast.cgi#alnHdr_625370251) | [AHY25151.1](http://www.ncbi.nlm.nih.gov/protein/625370251?report=genbank&log$=prottop&blast_rank=1&RID=T7PRD0RP015) | 64 | 100 | 1e -74 |
| 5 | + | ATG | 7077 8630 | 517 | portal vertex protein | [gp20 portal vertex protein of head [Aeromonas phage PX29]](http://blast.ncbi.nlm.nih.gov/blast/Blast.cgi#alnHdr_593774021) | [YP-009011644.1](http://www.ncbi.nlm.nih.gov/protein/593774021?report=genbank&log$=prottop&blast_rank=2&RID=T7R9BYX801R) | 57 | 99 | 0 |
| 6 | + | ATG | 8633 8803 | 56 | prohead core protein | [prohead core protein [Enterobacteria phage vB-EcoM-VR25]](http://blast.ncbi.nlm.nih.gov/blast/Blast.cgi#alnHdr_729030776) | [AIZ02529.1](http://www.ncbi.nlm.nih.gov/protein/729030776?report=genbank&log$=prottop&blast_rank=6&RID=T7RCH4WY01R) | 39 | 100 | 0.22 |
| 7 | + | ATG | 8803 9207 | 134 | prohead core protein | [putative prohead core protein [Cronobacter phage S13]](http://blast.ncbi.nlm.nih.gov/blast/Blast.cgi#alnHdr_641468778) | [AIA64882.1](http://www.ncbi.nlm.nih.gov/protein/641468778?report=genbank&log$=prottop&blast_rank=2&RID=T7SJWKJN01R) | 55 | 93 | 6e -31 |
| 8 | + | ATG | 9217 9852 | 211 | prohead core scaffolding | [gp21 prohead core scaffold protein and protease [Aeromonas phage PX29]](http://blast.ncbi.nlm.nih.gov/blast/Blast.cgi#alnHdr_593774024) | [YP-009011647.1](http://www.ncbi.nlm.nih.gov/protein/593774024?report=genbank&log$=prottop&blast_rank=2&RID=T7SMYEEJ01R) | 65 | 93 | 1e -80 |
| 9 | + | ATG | 9884 10684 | 266 | prohead core protein | [gp22 prohead core scaffold protein [Aeromonas phage Aeh1]](http://blast.ncbi.nlm.nih.gov/blast/Blast.cgi#alnHdr_38640156) | [NP-944112.1](http://www.ncbi.nlm.nih.gov/protein/38640156?report=genbank&log$=prottop&blast_rank=2&RID=T7T4V6M301R) | 53 | 99 | 2e -80 |
| 10 | + | ATG | 10740 11267 | 175 | major capsid protein | [gp23 major head protein [Aeromonas phage PX29]](http://blast.ncbi.nlm.nih.gov/blast/Blast.cgi#alnHdr_593774026) | [YP-009011649.1](http://www.ncbi.nlm.nih.gov/protein/593774026?report=genbank&log$=prottop&blast_rank=2&RID=T7T63ZZB01R) | 57 | 98 | 3e -65 |
| 11 | + | ATG | 11434 12324 | 296 | putative major capsid protein | [gp23 major head protein [Aeromonas phage PX29]](http://blast.ncbi.nlm.nih.gov/blast/Blast.cgi#alnHdr_593774026) | [YP-009011649.1](http://www.ncbi.nlm.nih.gov/protein/593774026?report=genbank&log$=prottop&blast_rank=3&RID=T7TNE1X501R) | 70 | 99 | 1e -145 |
|  |  |  | 12347 12366 |  | terminator | - | - | - | - | - |
| 12 | + | ATG | 12414 12764 | 116 | hypothetical protein | [hypothetical protein [Thioalkalivibrio sp. ALE12]](http://blast.ncbi.nlm.nih.gov/blast/Blast.cgi#alnHdr_740195277) | [WP-038037259.1](http://www.ncbi.nlm.nih.gov/protein/740195277?report=genbank&log$=prottop&blast_rank=3&RID=T7TRXT5901R) | 39 | 92 | 1e -13 |
|  |  |  | 12770 12793 |  | terminator | - | - | - | - | - |
| 13 | - | ATG | 12800 13375 | 191 | hypothetical protein | - | - | - | - | - |
| 14 | - | ATG | 13406 13984 | 192 | hypothetical protein | - | - | - | - | - |
| 15 | - | ATG | 14064 14477 | 137 | hypothetical protein | - | - | - | - | - |
| 16 | + | ATG | 14580 16091 | 503 | UvsW helicase | [UvsW RNA-DNA and DNA-DNA helicase/ATPase [Aeromonas phage PX29]](http://blast.ncbi.nlm.nih.gov/blast/Blast.cgi#alnHdr_593774042) | [YP-009011665.1](http://www.ncbi.nlm.nih.gov/protein/593774042?report=genbank&log$=prottop&blast_rank=2&RID=T7VNT8JK01R) | 61 | 99 | 0 |
|  |  |  | 16100 16119 |  | terminator | - | - | - | - | - |
| 17 | - | ATG | 16138 18027 | 629 | ADP-ribosyltransferase | [Alt RNA polymerase ADP-ribosylase [Aeromonas phage PX29]](http://blast.ncbi.nlm.nih.gov/blast/Blast.cgi#alnHdr_593774049) | [YP-009011672.1](http://www.ncbi.nlm.nih.gov/protein/593774049?report=genbank&log$=prottop&blast_rank=2&RID=T7X6575Z01R) | 35 | 98 | 1e -34 |
|  |  |  | 18077 18098 |  | terminator | - | - | - | - | - |
| 18 | - | ATG | 18103 18510 | 135 | Recombination, repair and ssDNA binding protein UvsY | [UvsY recombination, repair and single-stranded DNA binding protein [Aeromonas phage Aeh1]](http://blast.ncbi.nlm.nih.gov/blast/Blast.cgi#alnHdr_38640177) | [NP-944133.1](http://www.ncbi.nlm.nih.gov/protein/38640177?report=genbank&log$=prottop&blast_rank=3&RID=T7X7TW1P01R) | 41 | 99 | 2e -28 |
| 19 | - | ATG | 18507 18674 | 55 | hypothetical protein | [hypothetical protein CC31p188 [Enterobacteria phage CC31]](http://blast.ncbi.nlm.nih.gov/blast/Blast.cgi#alnHdr_311993180) | [YP-004010046.1](http://www.ncbi.nlm.nih.gov/protein/311993180?report=genbank&log$=prottop&blast_rank=1&RID=T7XTYB7J01R) | 47 | 96 | 4e -07 |
| 20 | - | ATG | 18703 19116 | 137 | hypothetical protein | [hypothetical protein [Selenomonas noxia]](http://blast.ncbi.nlm.nih.gov/blast/Blast.cgi#alnHdr_493747043) | [WP-006696062.1](http://www.ncbi.nlm.nih.gov/protein/493747043?report=genbank&log$=prottop&blast_rank=2&RID=T7XVCEER01R) | 35 | 97 | 0.023 |
| 21 | - | ATG | 19113 19376 | 87 | hypothetical protein | - | - | - | - | - |
| 22 | - | ATG | 19478 20758 | 426 | hypothetical protein | [Dda DNA helicase [Aeromonas phage Aeh1]](http://blast.ncbi.nlm.nih.gov/blast/Blast.cgi#alnHdr_38640180) | [NP-944136.1](http://www.ncbi.nlm.nih.gov/protein/38640180?report=genbank&log$=prottop&blast_rank=2&RID=T7YAKC0D01R) | 47 | 99 | 3e -126 |
| 23 | + | ATG | 20850 21998 | 382 | capsid vertex protein | [gp24 head vertex protein [Aeromonas phage 65]](http://blast.ncbi.nlm.nih.gov/blast/Blast.cgi#alnHdr_326536487) | [YP-004300918.1](http://www.ncbi.nlm.nih.gov/protein/326536487?report=genbank&log$=prottop&blast_rank=1&RID=T7YC62EU01R) | 35 | 97 | 3e -55 |
|  |  |  | 22005 22027 |  | terminator |  |  |  |  |  |
| 24 | - | ATG | 22032 22367 | 111 | hypothetical protein | [hypothetical protein VPHG-00154 [Vibrio phage 11895-B1]](http://blast.ncbi.nlm.nih.gov/blast/Blast.cgi#alnHdr_472340109) | [YP-007673637.1](http://www.ncbi.nlm.nih.gov/protein/472340109?report=genbank&log$=prottop&blast_rank=1&RID=T7ZCJJME01R) | 46 | 100 | 1e -18 |
| 25 | - | ATG | 22376 22819 | 147 | hypothetical protein | [-](http://blast.ncbi.nlm.nih.gov/blast/Blast.cgi#alnHdr_509141886) | - | - | - | - |
| 26 | - | ATG | 22858 23133 | 91 | hypothetical protein | - | - | - | - | - |
| 27 | - | ATG | 23187 24051 | 287 | hypothetical protein | [hypothetical protein RB43ORF241c [Enterobacteria phage RB43]](http://blast.ncbi.nlm.nih.gov/blast/Blast.cgi#alnHdr_66391692) | [YP-239217.1](http://www.ncbi.nlm.nih.gov/protein/66391692?report=genbank&log$=prottop&blast_rank=2&RID=T81K8MPB01R) | 57 | 98 | 3e -104 |
| 28 | - | ATG | 24088 24789 | 233 | N-formylglutamate deformylase | [N-formylglutamate amidohydrolase [Psychromonas sp. CNPT3]](http://blast.ncbi.nlm.nih.gov/blast/Blast.cgi#alnHdr_505278305) | [WP-015465407.1](http://www.ncbi.nlm.nih.gov/protein/505278305?report=genbank&log$=prottop&blast_rank=1&RID=T81MBC9V01R) | 57 | 98 | 8e -84 |
| 29 | - | ATG | 24802 25473 | 223 | hypothetical protein | - | - | - | - | - |
| 30 | - | ATG | 25502 25759 | 85 | hypothetical protein | - | - | - | - | - |
| 31 | - | ATG | 25818 26120 | 100 | hypothetical protein | - | - | - | - | - |
| 32 | - | ATG | 26113 26427 | 104 | hypothetical protein | - | - | - | - | - |
| 33 | - | ATG | 26438 26782 | 114 | hypothetical protein | [hypothetical protein ZX61-15000 [Vibrio sp. VPAP30]](http://blast.ncbi.nlm.nih.gov/blast/Blast.cgi#alnHdr_827525451) | [KLN64283.1](http://www.ncbi.nlm.nih.gov/protein/827525451?report=genbank&log$=prottop&blast_rank=1&RID=T8267AY901R) | 58 | 94 | 2e -35 |
|  |  |  | 26862 26881 |  | terminator | - | - | - | - | - |
| 34 | - | ATG | 27140 27649 | 169 | hypothetical protein | [hypothetical protein LPPPVgp01 [Listonella phage phiHSIC]](http://blast.ncbi.nlm.nih.gov/blast/Blast.cgi#alnHdr_62362364) | [YP-224229.1](http://www.ncbi.nlm.nih.gov/protein/62362364?report=genbank&log$=prottop&blast_rank=1&RID=T82GAJZF01R) | 58 | 100 | 1e -61 |
| 35 |  |  |  |  | tRNA-Met (cat) | - | - | - | - | - |
| 36 |  |  |  |  | tRNA-Arg (tct) | - | - | - | - | - |
| 37 |  |  |  |  | tRNA-Asn (gtt) | - | - | - | - | - |
| 38 | - | ATG | 28131 28607 | 158 | hypothetical protein | [hypothetical protein VPFG-00208 [Vibrio phage nt-1]](http://blast.ncbi.nlm.nih.gov/blast/Blast.cgi#alnHdr_514050583) | [YP-008125357.1](http://www.ncbi.nlm.nih.gov/protein/514050583?report=genbank&log$=prottop&blast_rank=1&RID=T82JU3S201R) | 48 | 96 | 4e -39 |
| 39 | - | ATG | 28788 28913 | 41 | hypothetical protein | - | - | - | - | - |
| 40 | - | TTG | 28910 29098 | 62 | hypothetical protein | - | - | - | - | - |
|  |  |  | 29135 29155 |  | terminator |  | - | - | - | - |
| 41 | - | ATG | 29169 30737 | 522 | hypothetical protein | - | - | - | - | - |
| 42 | - | ATG | 30823 31185 | 120 | hypothetical protein | [hypothetical protein VPHG-00175 [Vibrio phage 11895-B1]](http://blast.ncbi.nlm.nih.gov/blast/Blast.cgi#alnHdr_472340129) | [YP-007673657.1](http://www.ncbi.nlm.nih.gov/protein/472340129?report=genbank&log$=prottop&blast_rank=1&RID=T8CC119C01R) | 36 | 97 | 2e -15 |
| 43 | - | ATG | 31554 31742 | 62 | hypothetical protein | - | - | - | - | - |
| 44 | - | ATG | 31745 31924 | 59 | hypothetical protein | - | - | - | - | - |
| 45 | - | ATG | 31946 32194 | 82 | hypothetical protein | - | - | - | - | - |
| 46 | - | ATG | 32206 32412 | 68 | hypothetical protein | - | - | - | - | - |
| 47 | - | ATG | 32412 32660 | 82 | hypothetical protein | - | - | - | - | - |
| 48 | - | ATG | 32657 32890 | 77 | hypothetical protein | - | - | - | - | - |
| 49 | - | ATG | 33107 33370 | 87 | hypothetical protein | - | - | - | - | - |
| 50 | - | ATG | 33373 34116 | 247 | hypothetical protein | - | - | - | - | - |
| 51 | - | ATG | 34118 34354 | 78 | hypothetical protein | - | - | - | - | - |
|  |  |  | 34426 34445 |  | terminator | - | - | - | - | - |
| 52 | - | ATG | 34468 35430 | 320 | HEAT repeat | [hypothetical protein [Lactobacillus delbrueckii]](http://blast.ncbi.nlm.nih.gov/blast/Blast.cgi#alnHdr_737183776) | [WP-035169663.1](http://www.ncbi.nlm.nih.gov/protein/737183776?report=genbank&log$=prottop&blast_rank=1&RID=T8DC7EXE01R) | 46 | 72 | 4e -27 |
|  |  |  | 35499 35518 |  | terminator | - | - | - | - | - |
| 53 | - | ATG | 35526 35726 | 66 | hypothetical protein | - | - | - | - | - |
| 54 | - | ATG | 35710 36435 | 241 | hypothetical protein | [hypothetical protein VPLG-00041 [Vibrio phage eugene 12A10]](http://blast.ncbi.nlm.nih.gov/blast/Blast.cgi#alnHdr_510792857) | [AGN51480.1](http://www.ncbi.nlm.nih.gov/protein/510792857?report=genbank&log$=prottop&blast_rank=1&RID=T8DSXUAN01R) | 40 | 72 | 5e -31 |
| 55 | - | ATG | 36405 36617 | 70 | hypothetical protein | - | - | - | - | - |
| 56 | + | ATG | 37481 41263 | 1260 | long tail fiber, proximal subunit | [gp34 long tail fiber proximal subunit [Aeromonas phage Aeh1]](http://blast.ncbi.nlm.nih.gov/blast/Blast.cgi#alnHdr_38640255) | [NP-944211.1](http://www.ncbi.nlm.nih.gov/protein/38640255?report=genbank&log$=prottop&blast_rank=1&RID=T8ED308F01R) | 41 | 99 | 0 |
|  |  |  | 41284 41302 |  | terminator | - | - | - | - | - |
| 57 | + | ATG | 41346 44978 | 1210 | hypothetical protein | [-](http://blast.ncbi.nlm.nih.gov/blast/Blast.cgi#alnHdr_423261678) | - | - | -- | - |
| 58 | + | ATG | 45056 45484 | 142 | baseplate wedge subunit | [gp25 baseplate wedge subunit [Aeromonas phage 65]](http://blast.ncbi.nlm.nih.gov/blast/Blast.cgi#alnHdr_326536511) | [YP-004300942.1](http://www.ncbi.nlm.nih.gov/protein/326536511?report=genbank&log$=prottop&blast_rank=1&RID=T8EYWYT601R) | 48 | 85 | 2e -32 |
| 59 | + | ATG | 45497 47404 | 635 | baseplate wedge subunit | [gp6 baseplate wedge subunit [Aeromonas phage Aeh1]](http://blast.ncbi.nlm.nih.gov/blast/Blast.cgi#alnHdr_38640132) | [NP-944088.1](http://www.ncbi.nlm.nih.gov/protein/38640132?report=genbank&log$=prottop&blast_rank=1&RID=T8EH7UD501R) | 58 | 100 | 0 |
|  |  |  | 47422 47441 |  | terminator | - | - | - | - | - |
| 60 | - | ATG | 47450 48415 | 321 | SPFH domain / Band 7 family protein | [hypothetical protein [Photobacterium gaetbulicola]](http://blast.ncbi.nlm.nih.gov/blast/Blast.cgi#alnHdr_746420219) | [WP-039460904.1](http://www.ncbi.nlm.nih.gov/protein/746420219?report=genbank&log$=prottop&blast_rank=1&RID=T8EJPBN401R) | 61 | 93 | 1e -117 |
| 61 | - | ATG | 48420 48629 | 69 | hypothetical protein | - | - | - | - | - |
|  |  |  | 48654 48675 |  | terminator |  | - | - | - | - |
| 62 | - | ATG | 48689 49198 | 169 | hypothetical protein | - | - | - | - | - |
| 63 | - | ATG | 49211 49501 | 96 | hypothetical protein | - | - | - | - | - |
| 64 | - | ATG | 49582 50346 | 254 | hypothetical protein | - | - | - | - | - |
| 65 | - | ATG | 50330 50869 | 179 | N-acetylmuramoyl-L-alanine amidase CwlD | [N-acetylmuramoyl-L-alanine amidase [Vibrio phage phi 1]](http://blast.ncbi.nlm.nih.gov/blast/Blast.cgi#alnHdr_751186253) | [AJF40686.1](http://www.ncbi.nlm.nih.gov/protein/751186253?report=genbank&log$=prottop&blast_rank=4&RID=T8FRHUKX01R) | 44 | 96 | 9e -37 |
| 66 | - | GTG | 50881 51084 | 67 | hypothetical protein | - | - | - | - | - |
|  |  |  | 51123 51142 |  | terminator | - | - | - | - | - |
| 67 | - | ATG | 51163 52326 | 387 | hemin importer ATP-binding subunit | [hypothetical protein P12024S-24 [Persicivirga phage P12024S]](http://blast.ncbi.nlm.nih.gov/blast/Blast.cgi#alnHdr_399528443) | [YP-006560364.1](http://www.ncbi.nlm.nih.gov/protein/399528443?report=genbank&log$=prottop&blast_rank=1&RID=T8G22YGD01R) | 47 | 93 | 1e -114 |
| 68 | - | ATG | 52411 52758 | 115 | hypothetical protein | - | - | - | - | - |
| 69 | - | ATG | 52874 55051 | 725 | marine proteobacterial sortase target protein | [hypothetical protein CPTMiller-00120 [Citrobacter phage Miller]](http://blast.ncbi.nlm.nih.gov/blast/Blast.cgi#alnHdr_713322132) | [YP-009097722.1](http://www.ncbi.nlm.nih.gov/protein/713322132?report=genbank&log$=prottop&blast_rank=1&RID=T8GEAEKH01R) | 44 | 100 | 0 |
| 70 | - | ATG | 55114 55485 | 123 | hypothetical protein | - | - | - | - | - |
| 71 | - | ATG | 55498 55740 | 80 | hypothetical protein | - | - | - | - | - |
| 72 | + | ATG | 55855 56328 | 157 | hypothetical protein | [hypothetical protein S13-078 [Cronobacter phage S13]](http://blast.ncbi.nlm.nih.gov/blast/Blast.cgi#alnHdr_641468773) | [AIA64877.1](http://www.ncbi.nlm.nih.gov/protein/641468773?report=genbank&log$=prottop&blast_rank=2&RID=T8GTGH5N01R) | 40 | 84 | 1e -17 |
|  |  |  | 56333 56352 |  | terminator | - | - | - | - | - |
| 73 | - | ATG | 56378 56947 | 189 | hypothetical protein | - | - | - | - | - |
| 74 | - | ATG | 56944 57285 | 113 | hypothetical protein | - | - | - | - | - |
| 75 | - | ATG | 57630 57971 | 113 | hypothetical protein | - | - | - | - | - |
| 76 | - | ATG | 57975 58355 | 126 | hypothetical protein | - | - | - | - | - |
| 77 | - | ATG | 58378 58581 | 67 | hypothetical protein | [hypothetical protein [Vibrio cholerae]](http://blast.ncbi.nlm.nih.gov/blast/Blast.cgi#alnHdr_763261002) | [WP-044125605.1](http://www.ncbi.nlm.nih.gov/protein/763261002?report=genbank&log$=prottop&blast_rank=2&RID=T8HERAVW01R) | 52 | 89 | 1e -12 |
| 78 | - | ATG | 58616 59125 | 169 | hypothetical protein | [hypothetical protein LPPPVgp01 [Listonella phage phiHSIC]](http://blast.ncbi.nlm.nih.gov/blast/Blast.cgi#alnHdr_62362364) | [YP-224229.1](http://www.ncbi.nlm.nih.gov/protein/62362364?report=genbank&log$=prottop&blast_rank=1&RID=T8HFRB5N01R) | 64 | 100 | 1e -69 |
|  |  |  | 59334 59353 |  | terminator | - | - | - | - | - |
| 79 | - | ATG | 59367 61208 | 613 | DNA topoisomerase 4 subunit B | [topoisomerase II large subunit [Aeromonas phage Aeh1]](http://blast.ncbi.nlm.nih.gov/blast/Blast.cgi#alnHdr_38639928) | [NP-943883.1](http://www.ncbi.nlm.nih.gov/protein/38639928?report=genbank&log$=prottop&blast_rank=1&RID=T8HYYVV001R) | 55 | 97 | 0 |
| 80 | - | ATG | 61211 61405 | 64 | hypothetical protein | - | - | - | - | - |
| 81 | - | ATG | 61436 62113 | 225 | exonuclease | [putative exonuclease A [Cronobacter phage S13]](http://blast.ncbi.nlm.nih.gov/blast/Blast.cgi#alnHdr_641468950) | [AIA65054.1](http://www.ncbi.nlm.nih.gov/protein/641468950?report=genbank&log$=prottop&blast_rank=1&RID=T8JG7BCJ01R) | 45 | 95 | 7e -61 |
| 82 | - | ATG | 62178 62795 | 205 | Deoxyuridine 5'-triphosphate nucleotidohydrolase | [deoxyuridine 5'-triphosphate nucleotidohydrolase [Serratia liquefaciens]](http://blast.ncbi.nlm.nih.gov/blast/Blast.cgi#alnHdr_526015803) | [WP-020837636.1](http://www.ncbi.nlm.nih.gov/protein/526015803?report=genbank&log$=prottop&blast_rank=2&RID=T8JZU53U01R) | 45 | 77 | 1e -32 |
| 83 | - | ATG | 62878 63705 | 275 | Phosphate starvation-inducible protein PsiH | [phosphate starvation protein PhoH [Methylobacterium sp. B34]](http://blast.ncbi.nlm.nih.gov/blast/Blast.cgi#alnHdr_757119168) | [WP-042673543.1](http://www.ncbi.nlm.nih.gov/protein/757119168?report=genbank&log$=prottop&blast_rank=1&RID=T8JJ9Y0R01R) | 35 | 85 | 6e -28 |
| 84 | - | ATG | 63690 64739 | 349 | DNA primase | [gp61 DNA primase subunit [Aeromonas phage PX29]](http://blast.ncbi.nlm.nih.gov/blast/Blast.cgi#alnHdr_593773816) | [YP-009011439.1](http://www.ncbi.nlm.nih.gov/protein/593773816?report=genbank&log$=prottop&blast_rank=1&RID=T8K2HTG701R) | 49 | 97 | 2e -106 |
|  |  |  | 64809 64827 |  | terminator | - | - | - | - | - |
| 85 | - | ATG | 64843 64956 | 37 | hypothetical protein | - | - | - | - | - |
| 86 | - | ATG | 65064 65552 | 162 | hypothetical protein | [hypothetical protein [Streptomyces ahygroscopicus]](http://blast.ncbi.nlm.nih.gov/blast/Blast.cgi#alnHdr_764447725) | [WP-044370231.1](http://www.ncbi.nlm.nih.gov/protein/764447725?report=genbank&log$=prottop&blast_rank=2&RID=T8KCX93F01R) | 42 | 90 | 3e -29 |
| 87 | - | TTG | 65594 66760 | 388 | 41 helicase | [gp41 DNA primase-helicase subunit [Aeromonas phage Aeh1]](http://blast.ncbi.nlm.nih.gov/blast/Blast.cgi#alnHdr_38639936) | [NP-943891.1](http://www.ncbi.nlm.nih.gov/protein/38639936?report=genbank&log$=prottop&blast_rank=1&RID=T8KDVRT101R) | 58 | 70 | 2e -112 |
| 88 | - | ATG | 66732 67868 | 378 | hypothetical protein | - | - | - | - | - |
| 89 | - | ATG | 67971 68591 | 206 | 41 helicase | [gp41 DNA primase-helicase subunit [Aeromonas phage Aeh1]](http://blast.ncbi.nlm.nih.gov/blast/Blast.cgi#alnHdr_38639936) | [NP-943891.1](http://www.ncbi.nlm.nih.gov/protein/38639936?report=genbank&log$=prottop&blast_rank=1&RID=T8KZV38T01R) | 58 | 88 | 2e -71 |
| 90 | - | ATG | 68540 68923 | 127 | hypothetical protein | - | - | - | - | - |
| 91 | - | ATG | 69004 69555 | 183 | hypothetical protein | [RecA-like recombination protein [Yersinia phage vB-YenM-TG1]](http://blast.ncbi.nlm.nih.gov/blast/Blast.cgi#alnHdr_746946423) | [AJD81851.1](http://www.ncbi.nlm.nih.gov/protein/746946423?report=genbank&log$=prottop&blast_rank=5&RID=T8MKXRAK01R) | 52 | 98 | 6e -63 |
| 92 | - | ATG | 69663 70064 | 133 | hypothetical protein | [homing endonuclease [Proteus phage vB-PmiM-Pm5461]](http://blast.ncbi.nlm.nih.gov/blast/Blast.cgi#alnHdr_797193776) | [AKA61894.1](http://www.ncbi.nlm.nih.gov/protein/797193776?report=genbank&log$=prottop&blast_rank=1&RID=T8N9U4N601R) | 42 | 87 | 2e -15 |
| 93 | - | ATG | 70057 70971 | 304 | hypothetical protein | [RecA-like recombination protein [Proteus phage vB-PmiM-Pm5461]](http://blast.ncbi.nlm.nih.gov/blast/Blast.cgi#alnHdr_797193777) | [AKA61895.1](http://www.ncbi.nlm.nih.gov/protein/797193777?report=genbank&log$=prottop&blast_rank=1&RID=T8MNS1U101R) | 62 | 99 | 3e -137 |
| 94 | - | ATG | 71058 71663 | 201 | DNA polymerase | [putative DNA polymerase [Cronobacter phage S13]](http://blast.ncbi.nlm.nih.gov/blast/Blast.cgi#alnHdr_641468943) | [AIA65047.1](http://www.ncbi.nlm.nih.gov/protein/641468943?report=genbank&log$=prottop&blast_rank=1&RID=T8NJE6Z001R) | 52 | 100 | 9e -61 |
| 95 | - | ATG | 71752 72627 | 291 | DNA polymerase | [putative DNA polymerase [Cronobacter phage S13]](http://blast.ncbi.nlm.nih.gov/blast/Blast.cgi#alnHdr_641468943) | [AIA65047.1](http://www.ncbi.nlm.nih.gov/protein/641468943?report=genbank&log$=prottop&blast_rank=1&RID=T8MS9AUE01R) | 57 | 70 | 1e -57 |
| 96 | - | ATG | 72662 73198 | 178 | hypothetical protein | [hypothetical protein NEIFL0001-0538 [Neisseria flavescens SK114]](http://blast.ncbi.nlm.nih.gov/blast/Blast.cgi#alnHdr_241319801) | [EER56197.1](http://www.ncbi.nlm.nih.gov/protein/241319801?report=genbank&log$=prottop&blast_rank=1&RID=T8NU394401R) | 47 | 98 | 7e -46 |
|  |  |  | 73245 73267 |  | terminator | - | - | - | - | - |
| 97 | - | ATG | 73277 73600 | 107 | DNA polymerase | [gp43A core DNA polymerase of replisome [Aeromonas phage 65]](http://blast.ncbi.nlm.nih.gov/blast/Blast.cgi#alnHdr_326536431) | [YP-004300861.1](http://www.ncbi.nlm.nih.gov/protein/326536431?report=genbank&log$=prottop&blast_rank=1&RID=T8NV46G501R) | 58 | 98 | 1e -30 |
| 98 | - | ATG | 73795 74619 | 274 | hypothetical protein | [putative intron I-LimII [Dickeya phage Limestone]](http://blast.ncbi.nlm.nih.gov/blast/Blast.cgi#alnHdr_448260124) | [YP-007237501.1](http://www.ncbi.nlm.nih.gov/protein/448260124?report=genbank&log$=prottop&blast_rank=1&RID=T8P3NE9D01R) | 36 | 85 | 3e -30 |
| 99 | - | ATG | 74775 75374 | 199 | DNA polymerase | [DNA polymerase [Aeromonas phage Aeh1]](http://blast.ncbi.nlm.nih.gov/blast/Blast.cgi#alnHdr_38639940) | [NP-943895.1](http://www.ncbi.nlm.nih.gov/protein/38639940?report=genbank&log$=prottop&blast_rank=1&RID=T8P503XH01R) | 60 | 92 | 5e -65 |
| 100 | - | ATG | 75451 76035 | 194 | DNA polymerase | [DNA polymerase [Aeromonas phage Aeh1]](http://blast.ncbi.nlm.nih.gov/Blast.cgi#alnHdr_38639940) | NP 943895.1 | 50 | 99 | 3e -49 |
|  |  |  | 76106 76124 |  | terminator | - | - | - | - | - |
| 101 | - | ATG | 76140 77084 | 314 | single-stranded DNA binding protein | [gp32 single-stranded DNA binding protein [Aeromonas phage Aeh1]](http://blast.ncbi.nlm.nih.gov/Blast.cgi#alnHdr_38640250) | [NP-944206.1](http://www.ncbi.nlm.nih.gov/protein/38640250?report=genbank&log$=prottop&blast_rank=2&RID=T9NG99SX014) | 61 | 100 | 4e -131 |
| 102 | - | ATG | 77329 77997 | 222 | 59 protein | [gp59 loader of gp41 DNA helicase [Aeromonas phage PX29]](http://blast.ncbi.nlm.nih.gov/Blast.cgi#alnHdr_593774113) | [YP-009011736.1](http://www.ncbi.nlm.nih.gov/protein/593774113?report=genbank&log$=prottop&blast_rank=1&RID=T9N8NN4U015) | 44 | 97 | 2e -69 |
| 103 | - | ATG | 78002 78250 | 82 | hypothetical protein | [-](http://blast.ncbi.nlm.nih.gov/Blast.cgi#alnHdr_589891984) | - | - | - | - |
| 104 | - | ATG | 78237 78530 | 97 | double-stranded DNA binding protein | [putative dsDNA binding protein [Cronobacter phage S13]](http://blast.ncbi.nlm.nih.gov/Blast.cgi#alnHdr_641468707) | [AIA64811.1](http://www.ncbi.nlm.nih.gov/protein/641468707?report=genbank&log$=prottop&blast_rank=1&RID=T9P121RN015) | 46 | 82 | 3e -15 |
| 105 | - | ATG | 78592 79548 | 318 | RnaseH | [RnaseH ribonuclease [Enterobacteria phage RB49]](http://blast.ncbi.nlm.nih.gov/Blast.cgi#alnHdr_33620629) | [NP-891817.1](http://www.ncbi.nlm.nih.gov/protein/33620629?report=genbank&log$=prottop&blast_rank=1&RID=T9P8T5U3015) | 41 | 98 | 1e -85 |
| 106 | + | ATG | 79631 79930 | 99 | hypothetical protein | - | - | - | - | - |
|  |  |  | 79938 79957 |  | terminator | - | - | - | - | - |
| 107 | - | ATG | 79947 80624 | 225 | hypothetical protein | [gp17 terminase DNA packaging enzyme large subunit [Aeromonas phage Aeh1]](http://blast.ncbi.nlm.nih.gov/Blast.cgi#alnHdr_38640149) | [NP-944105.1](http://www.ncbi.nlm.nih.gov/protein/38640149?report=genbank&log$=prottop&blast_rank=2&RID=T9PBJ0AK014) | 51 | 84 | 1e -60 |
| 108 | - | ATG | 80621 81091 | 156 | hypothetical protein | - | - | - | - | - |
| 109 | - | ATG | 81078 81587 | 169 | small terminase protein | [gp16 terminase DNA packaging enzyme small subunit [Acinetobacter phage Ac42]](http://blast.ncbi.nlm.nih.gov/Blast.cgi#alnHdr_311992669) | [YP-004009537.1](http://www.ncbi.nlm.nih.gov/protein/311992669?report=genbank&log$=prottop&blast_rank=2&RID=T9PNX71K014) | 43 | 94 | 1e -30 |
| 110 | - | ATG | 81580 82317 | 245 | tail sheath stabilizer and completion protein | [tail sheath stabilizer and completion protein [Enterobacteria phage JSE]](http://blast.ncbi.nlm.nih.gov/Blast.cgi#alnHdr_238695033) | [YP-002922227.1](http://www.ncbi.nlm.nih.gov/protein/238695033?report=genbank&log$=prottop&blast_rank=2&RID=T9PWEFK9015) | 45 | 98 | 1e -60 |
| 111 | - | ATG | 82322 83230 | 302 | baseplate subunit | [gp54 baseplate tail tube initiator [Aeromonas phage Aeh1]](http://blast.ncbi.nlm.nih.gov/Blast.cgi#alnHdr_38640122) | [NP-944078.1](http://www.ncbi.nlm.nih.gov/protein/38640122?report=genbank&log$=prottop&blast_rank=1&RID=T9PY3XFC015) | 45 | 79 | 5e -60 |
| 112 | - | ATG | 83268 83567 | 99 | DNA end protector protein | [gp2 DNA end protector protein [Aeromonas phage Aeh1]](http://blast.ncbi.nlm.nih.gov/Blast.cgi#alnHdr_38640123) | [NP-944079.1](http://www.ncbi.nlm.nih.gov/protein/38640123?report=genbank&log$=prottop&blast_rank=3&RID=T9R8RAX9014) | 64 | 100 | 1e -38 |
| 113 | - | ATG | 83593 84282 | 229 | DNA end protector protein | [gp2 DNA end protector protein [Aeromonas phage Aeh1]](http://blast.ncbi.nlm.nih.gov/Blast.cgi#alnHdr_38640123) | [NP-944079.1](http://www.ncbi.nlm.nih.gov/protein/38640123?report=genbank&log$=prottop&blast_rank=2&RID=T9RETDXT015) | 46 | 96 | 4e -61 |
| 114 | - | ATG | 84282 84887 | 201 | hypothetical protein | [hypothetical protein LPPPVgp01 [Listonella phage phiHSIC]](http://blast.ncbi.nlm.nih.gov/Blast.cgi#alnHdr_62362364) | [YP-224229.1](http://www.ncbi.nlm.nih.gov/protein/62362364?report=genbank&log$=prottop&blast_rank=1&RID=T9RMEBRC014) | 57 | 99 | 3e -75 |
| 115 | - | ATG | 84951 85322 | 123 | head completion protein | [head completion protein [Proteus phage vB-PmiM-Pm5461]](http://blast.ncbi.nlm.nih.gov/Blast.cgi#alnHdr_797193879) | AKA61997.1 | 57 | 97 | 2e -37 |
| 116 | + | ATG | 85462 86460 | 332 | Tail-tube assembly protein | [gp48 baseplate tail tube cap [Aeromonas phage 65]](http://blast.ncbi.nlm.nih.gov/Blast.cgi#alnHdr_326536519) | [YP-004300950.1](http://www.ncbi.nlm.nih.gov/protein/326536519?report=genbank&log$=prottop&blast_rank=1&RID=T9RW3YSV014) | 49 | 92 | 1e -95 |
| 117 | + | ATG | 86460 87011 | 183 | baseplate wedge subunit | [baseplate wedge subunit [Aeromonas phage CC2]](http://blast.ncbi.nlm.nih.gov/Blast.cgi#alnHdr_423261830) | [YP-007010366.1](http://www.ncbi.nlm.nih.gov/protein/423261830?report=genbank&log$=prottop&blast_rank=1&RID=T9RXYCTA014) | 49 | 96 | 6e -56 |
|  |  |  | 87016 87033 |  | terminator | - | - | - | - | - |
| 118 | - | ATG | 87046 87294 | 82 | hypothetical protein | - | - | - | - | - |
| 119 | - | ATG | 87365 88063 | 232 | deoxynucleoside monophosphate kinase | [dNMP kinase [Citrobacter phage Miller]](http://blast.ncbi.nlm.nih.gov/Blast.cgi#alnHdr_713322204) | [YP-009097794.1](http://www.ncbi.nlm.nih.gov/protein/713322204?report=genbank&log$=prottop&blast_rank=1&RID=T9SRJRXV015) | 35 | 90 | 2e -20 |
| 120 | - | ATG | 88149 88604 | 151 | tail completion and sheath stabilizer protein | [tail completion and sheath stabilizer protein [Aeromonas phage CC2]](http://blast.ncbi.nlm.nih.gov/Blast.cgi#alnHdr_423261836) | [YP-007010373.1](http://www.ncbi.nlm.nih.gov/protein/423261836?report=genbank&log$=prottop&blast_rank=1&RID=T9SSTJSE014) | 36 | 94 | 1e -12 |
| 121 | - | ATG | 88606 90300 | 564 | hypothetical protein | [-](http://blast.ncbi.nlm.nih.gov/Blast.cgi#alnHdr_326536526) | - | - | - | - |
| 122 | - | ATG | 90514 91272 | 252 | T4 bacteriophage base plate protein | [gp26 baseplate hub subunit [Aeromonas phage Aeh1]](http://blast.ncbi.nlm.nih.gov/Blast.cgi#alnHdr_38640121) | NP-944077.1 | 39 | 98 | 7e -64 |
| 123 | - | GTG | 91274 92125 | 283 | hypothetical protein | - | - | - | - | - |
| 124 | - | ATG | 92125 92619 | 164 | hypothetical protein | [-](http://blast.ncbi.nlm.nih.gov/Blast.cgi#alnHdr_593773983) | - | - | - | - |
| 125 | + | ATG | 93338 95716 | 792 | Ribonucleoside-diphosphate reductase 1 subunit alpha | [aerobic ribonucleoside diphosphate reductase large subunit [Vibrio phage KVP40]](http://blast.ncbi.nlm.nih.gov/Blast.cgi#alnHdr_34419510) | [NP-899523.1](http://www.ncbi.nlm.nih.gov/protein/34419510?report=genbank&log$=prottop&blast_rank=1&RID=T9U3TSSV014) | 58 | 100 | 0 |
| 126 | + | ATG | 95804 96907 | 367 | Ribonucleoside-diphosphate reductase 1 subunit beta | [ribonucleoside-diphosphate reductase 1 subunit beta [Vibrio phage VH7D]](http://blast.ncbi.nlm.nih.gov/Blast.cgi#alnHdr_589286439) | [YP-009006237.1](http://www.ncbi.nlm.nih.gov/protein/589286439?report=genbank&log$=prottop&blast_rank=1&RID=T9U70WWA014) | 62 | 99 | 4e -172 |
| 127 | + | ATG | 96923 97186 | 87 | glutaredoxin 1 | [glutaredoxin [Vibrio phage VH7D]](http://blast.ncbi.nlm.nih.gov/Blast.cgi#alnHdr_589286440) | [YP-009006238.1](http://www.ncbi.nlm.nih.gov/protein/589286440?report=genbank&log$=prottop&blast_rank=1&RID=T9UKV675015) | 40 | 94 | 7e -12 |
|  |  |  | 97234 97258 |  | terminator | - | - | - | - | - |
| 128 | + | ATG | 97287 99422 | 711 | Anaerobic ribonucleoside-triphosphate reductase | [ribonucleoside triphosphate reductase [Vibrio furnissii]](http://blast.ncbi.nlm.nih.gov/Blast.cgi#alnHdr_490866174) | [WP-004728196.1](http://www.ncbi.nlm.nih.gov/protein/490866174?report=genbank&log$=prottop&blast_rank=1&RID=T9UNBZSD014) | 55 | 99 | 0 |
| 129 | + | TTG | 99412 99897 | 161 | anaerobic ribonucleotide reductase-activating protein | [anaerobic NTP reductase small subunit [Aeromonas phage CC2]](http://blast.ncbi.nlm.nih.gov/Blast.cgi#alnHdr_423261795) | [YP-007010295.1](http://www.ncbi.nlm.nih.gov/protein/423261795?report=genbank&log$=prottop&blast_rank=1&RID=T9UPYD2J014) | 53 | 90 | 2e -44 |
| 130 | + | ATG | 99913 100101 | 62 | hypothetical protein | - | - | - | - | - |
|  |  |  | 100126 100149 |  | terminator |  | - | - | - | - |
| 131 | + | TTG | 100154 100378 | 74 | hypothetical protein | - | - | - | - | - |
| 132 | + | ATG | 100414 100728 | 104 | hypothetical protein | - | - | - | - | - |
| 133 | + | ATG | 100721 101251 | 176 | hypothetical protein | [hypothetical protein CC2-265 [Aeromonas phage CC2]](http://blast.ncbi.nlm.nih.gov/Blast.cgi#alnHdr_423261674) | [YP-007010291.1](http://www.ncbi.nlm.nih.gov/protein/423261674?report=genbank&log$=prottop&blast_rank=2&RID=T9WPCPYT015) | 43 | 92 | 3e -34 |
| 134 | + | TTG | 101254 101514 | 86 | hypothetical protein | - | - | - | - | - |
| 135 | + | ATG | 101523 102953 | 476 | hypothetical protein | [hypothetical protein Aeh1p034 [Aeromonas phage Aeh1]](http://blast.ncbi.nlm.nih.gov/Blast.cgi#alnHdr_38639957) | [NP-943912.1](http://www.ncbi.nlm.nih.gov/protein/38639957?report=genbank&log$=prottop&blast_rank=1&RID=T9X3077X014) | 43 | 99 | 6e -126 |
| 136 | + | ATG | 103024 103608 | 194 | RNA polymerase sigma factor | [sigma factor [Acinetobacter phage ZZ1]](http://blast.ncbi.nlm.nih.gov/Blast.cgi#alnHdr_392972877) | [YP-006488835.1](http://www.ncbi.nlm.nih.gov/protein/392972877?report=genbank&log$=prottop&blast_rank=1&RID=T9X4TD03015) | 41 | 85 | 1e -34 |
| 137 | + | ATG | 103614 103952 | 112 | hypothetical protein | [hypothetical protein pp2-067 [Vibriophage phi-pp2]](http://blast.ncbi.nlm.nih.gov/Blast.cgi#alnHdr_394774628) | [AFN37300.1](http://www.ncbi.nlm.nih.gov/protein/394774628?report=genbank&log$=prottop&blast_rank=1&RID=T9ZMF2SA015) | 38 | 80 | 4e -11 |
|  |  |  | 103959 103982 |  | terminator | - | - | - | - | - |
| 138 | + | ATG | 104033 105058 | 342 | endonuclease subunit | [gp47 recombination endonuclease subunit [Aeromonas phage PX29]](http://blast.ncbi.nlm.nih.gov/Blast.cgi#alnHdr_593773830) | [YP-009011453.1](http://www.ncbi.nlm.nih.gov/protein/593773830?report=genbank&log$=prottop&blast_rank=2&RID=T9ZNTRDY015) | 50 | 98 | 2e -106 |
| 139 | + | ATG | 105064 107448 | 794 | endonuclease subunit | [gp46 recombination endonuclease subunit [Aeromonas phage PX29]](http://blast.ncbi.nlm.nih.gov/Blast.cgi#alnHdr_593773829) | [YP-009011452.1](http://www.ncbi.nlm.nih.gov/protein/593773829?report=genbank&log$=prottop&blast_rank=1&RID=TA07APED014) | 35 | 99 | 2e -141 |
| 140 | + | ATG | 107626 108228 | 200 | hypothetical protein | [hypothetical protein LPPPVgp01 [Listonella phage phiHSIC]](http://blast.ncbi.nlm.nih.gov/Blast.cgi#alnHdr_62362364) | [YP-224229.1](http://www.ncbi.nlm.nih.gov/protein/62362364?report=genbank&log$=prottop&blast_rank=1&RID=T9ZT37J3015) | 58 | 99 | 2e -70 |
| 141 | + | ATG | 108264 108461 | 65 | hypothetical protein | - | - | - | - | - |
| 142 | + | ATG | 108436 109095 | 219 | sliding clamp | [gp45 sliding clamp protein [Aeromonas phage PX29]](http://blast.ncbi.nlm.nih.gov/Blast.cgi#alnHdr_593773825) | [YP-009011448.1](http://www.ncbi.nlm.nih.gov/protein/593773825?report=genbank&log$=prottop&blast_rank=3&RID=TA0N7CM9015) | 35 | 99 | 1e -31 |
|  |  |  | 109105 109129 |  | terminator | - | - | - | - | - |
| 143 | + | ATG | 109176 110150 | 324 | clamp loader, small subunit | [gp44 clamp loader subunit [Aeromonas phage PX29]](http://blast.ncbi.nlm.nih.gov/Blast.cgi#alnHdr_593773824) | [YP-009011447.1](http://www.ncbi.nlm.nih.gov/protein/593773824?report=genbank&log$=prottop&blast_rank=1&RID=TA0PCCGG01R) | 61 | 99 | 1e -144 |
| 144 | + | ATG | 110147 110734 | 195 | clamp loader, small subunit | [gp62 clamp loader subunit [Aeromonas phage Aeh1]](http://blast.ncbi.nlm.nih.gov/Blast.cgi#alnHdr_38639942) | [NP-943897.1](http://www.ncbi.nlm.nih.gov/protein/38639942?report=genbank&log$=prottop&blast_rank=1&RID=TA0Y8UGZ014) | 48 | 98 | 3e -55 |
| 145 | + | ATG | 110766 111167 | 133 | translation repressor protein | [translational repressor protein [Serratia phage PS2]](http://blast.ncbi.nlm.nih.gov/Blast.cgi#alnHdr_639438368) | [YP-009030100.1](http://www.ncbi.nlm.nih.gov/protein/639438368?report=genbank&log$=prottop&blast_rank=2&RID=TA3MC8F8014) | 60 | 89 | 2e -42 |
| 146 | + | ATG | 111160 111735 | 191 | Dihydrofolate reductase | [dihydrofolate reductase [Enterobacteria phage RB3]](http://blast.ncbi.nlm.nih.gov/Blast.cgi#alnHdr_713322535) | [YP-009098619.1](http://www.ncbi.nlm.nih.gov/protein/713322535?report=genbank&log$=prottop&blast_rank=2&RID=TA103C7W014) | 35 | 98 | 1e -19 |
| 147 | + | ATG | 111738 111989 | 83 | hypothetical protein | - | - | - | - | - |
| 148 | + | TTG | 112008 113231 | 407 | Thymidylate synthase | [Putative dTMP thymidylate synthase [Enterobacteria phage GEC-3S]](http://blast.ncbi.nlm.nih.gov/Blast.cgi#alnHdr_754380834) | [YP-009118920.1](http://www.ncbi.nlm.nih.gov/protein/754380834?report=genbank&log$=prottop&blast_rank=1&RID=TA3W6AEF014) | 40 | 100 | 1e -78 |
|  |  |  | 113245 113271 |  | terminator |  | - | - | - | - |
| 149 | + | ATG | 113398 113607 | 69 | hypothetical protein | - | - | - | - | - |
| 150 | + | ATG | 113673 113861 | 62 | hypothetical protein | - | - | - | - | - |
| 151 | + | ATG | 113961 114461 | 166 | hypothetical protein | - | - | - | - | - |
| 152 | + | ATG | 114490 114990 | 166 | hypothetical protein | [hypothetical protein CC2-102 [Aeromonas phage CC2]](http://blast.ncbi.nlm.nih.gov/Blast.cgi#alnHdr_423261616) | YP-007010128.1 | 37 | 99 | 3e -16 |
| 153 | + | ATG | 115012 115302 | 96 | hypothetical protein | - | - | - | - | - |
| 154 | + | TTG | 115395 116447 | 350 | hypothetical protein | [DnaJ domain-containing protein [Escherichia phage Lw1]](http://blast.ncbi.nlm.nih.gov/Blast.cgi#alnHdr_509141718) | [YP-008060583.1](http://www.ncbi.nlm.nih.gov/protein/509141718?report=genbank&log$=prottop&blast_rank=2&RID=TA570NZD015) | 35 | 92 | 1e -28 |
|  |  |  | 116460 116479 |  | terminator | - | - | - | - | - |
| 155 | + | GTG | 116546 117007 | 153 | hypothetical protein | [hypothetical protein VPIG-00017 [Vibrio phage PWH3a-P1]](http://blast.ncbi.nlm.nih.gov/Blast.cgi#alnHdr_472342369) | [YP-007675877.1](http://www.ncbi.nlm.nih.gov/protein/472342369?report=genbank&log$=prottop&blast_rank=1&RID=TA5WZ9DJ014) | 43 | 100 | 1e -28 |
|  |  |  | 117014 117031 |  | terminator | - | - | - | - | - |
| 156 | + | ATG | 117099 117518 | 139 | hypothetical protein | - | - | - | - | - |
| 157 | - | ATG | 117531 119615 | 694 | putative protein related to plant photosystem II stability/assembly factor | - | - | - | - | - |
| 158 | - | ATG | 119602 119832 | 76 | hypothetical protein | - | - | - | - | - |
|  |  |  | 119880 119901 |  | terminator |  | - | -- | - | - |
| 159 | - | ATG | 119908 121476 | 522 | hypothetical protein | - | - | - | - | - |
| 160 | - | ATG | 121476 123887 | 803 | hypothetical protein | - | - | - | - | - |
| 161 | - | TTG | 123895 125787 | 630 | hypothetical protein | - | - | - | - | - |
|  |  |  | 125838 125859 |  | terminator |  | - | - | - | - |
| 162 | - | ATG | 125845 126153 | 102 | hypothetical protein | [pseT.2 gene product [Enterobacteria phage IME08]](http://blast.ncbi.nlm.nih.gov/Blast.cgi#alnHdr_299779169) | YP-003734363.1 | 36 | 92 | 3e -07 |
| 163 | - | ATG | 126141 126527 | 128 | hypothetical protein | [hypothetical protein S13-030 [Cronobacter phage S13]](http://blast.ncbi.nlm.nih.gov/Blast.cgi#alnHdr_641468725) | [AIA64829.1](http://www.ncbi.nlm.nih.gov/protein/641468725?report=genbank&log$=prottop&blast_rank=1&RID=TA8WXTR1014) | 35 | 94 | 3e -09 |
|  |  |  | 126557 126576 |  | terminator | - | - | - | - | - |
| 164 | - | ATG | 126600 127049 | 149 | hypothetical protein | [hypothetical protein YenMTG1-114 [Yersinia phage vB-YenM-TG1]](http://blast.ncbi.nlm.nih.gov/Blast.cgi#alnHdr_746946496) | [AJD81924.1](http://www.ncbi.nlm.nih.gov/protein/746946496?report=genbank&log$=prottop&blast_rank=1&RID=TA9497N4014) | 46 | 96 | 4e -26 |
| 165 | - | ATG | 127234 128292 | 352 | Bifunctional NMN adenylyltransferase/Nudix hydrolase | [conserved hypothetical protein [Aeromonas phage PX29]](http://blast.ncbi.nlm.nih.gov/Blast.cgi#alnHdr_593774084) | [YP-009011707.1](http://www.ncbi.nlm.nih.gov/protein/593774084?report=genbank&log$=prottop&blast_rank=1&RID=TA8Z3ZEC014) | 49 | 99 | 1e -111 |
| 166 | - | TTG | 128359 129486 | 375 | RNA ligase A | [RNA ligase 1 [Citrobacter phage Moon]](http://blast.ncbi.nlm.nih.gov/Blast.cgi#alnHdr_723006479) | [IX12216.1](http://www.ncbi.nlm.nih.gov/protein/723006479?report=genbank&log$=prottop&blast_rank=1&RID=TA9R00WC015) | 41 | 97 | 2e -80 |
| 167 | - | ATG | 129473 129844 | 123 | endonuclease II | [DenA endonuclease II [Aeromonas phage Aeh1]](http://blast.ncbi.nlm.nih.gov/Blast.cgi#alnHdr_38640242) | NP-944198.1 | 43 | 95 | 2e -23 |
| 168 | - | ATG | 129928 130527 | 199 | hypothetical protein | - | - | - | - | - |
| 169 | - | ATG | 130597 130770 | 57 | hypothetical protein | - | - | - | - | - |
| 170 | - | ATG | 130780 130905 | 41 | hypothetical protein | - | - | - | - | - |
| 171 | - | ATG | 130911 131327 | 138 | hypothetical protein | - | - | - | - | - |
|  |  |  | 131398 131417 |  |  |  | - | - | - | - |
| 172 | - | ATG | 131426 131662 | 78 | hypothetical protein | [hypothetical protein VPIG-00020 [Vibrio phage PWH3a-P1]](http://blast.ncbi.nlm.nih.gov/Blast.cgi#alnHdr_472342372) | [YP-007675880.1](http://www.ncbi.nlm.nih.gov/protein/472342372?report=genbank&log$=prottop&blast_rank=1&RID=TAACXCCZ014) | 35 | 94 | 1e -06 |
|  |  |  | 131800 131819 |  | terminator | - | - | - | - | - |
| 173 | - | ATG | 131827 132042 | 71 | hypothetical protein | - | - | - | - | - |
| 174 | - | ATG | 132240 132506 | 88 | hypothetical protein | - | - | - | - | - |
| 175 | - | ATG | 132636 132866 | 76 | hypothetical protein | - | - | - | - | - |
| 176 | - | ATG | 132994 133266 | 90 | hypothetical protein | [hypothetical protein VPBG-00095 [Vibrio phage helene 12B3]](http://blast.ncbi.nlm.nih.gov/Blast.cgi#alnHdr_481018899) | [YP-007877256.1](http://www.ncbi.nlm.nih.gov/protein/481018899?report=genbank&log$=prottop&blast_rank=2&RID=TAATC2CM015) | 37 | 86 | 1e -12 |
| 177 | - | ATG | 133328 133549 | 73 | hypothetical protein | - | - | - | - | - |
|  |  |  | 133627 133646 |  | terminator | - | - | - | - | - |
| 178 | - | ATG | 133659 133949 | 96 | hypothetical protein | - | - | - | - | - |
| 179 | + | ATG | 134125 134349 | 74 | hypothetical protein | - | - | - | - | - |
| 180 | - | ATG | 134532 134768 | 78 | hypothetical protein | - | - | - | - | - |
| 181 | - | ATG | 134758 135756 | 332 | Multifunctional CCA protein | [multifunctional CCA protein [Vibrio parahaemolyticus]](http://blast.ncbi.nlm.nih.gov/Blast.cgi#alnHdr_545124052) | [WP-021485515.1](http://www.ncbi.nlm.nih.gov/protein/545124052?report=genbank&log$=prottop&blast_rank=2&RID=TABA8S2C014) | 51 | 86 | 2e -49 |
| 182 | - | ATG | 135735 136457 | 240 | hypothetical protein | - | - | - | - | - |
| 183 | - | ATG | 136454 136744 | 96 | hypothetical protein | - | - | - | - | - |
| 184 | - | ATG | 136686 137018 | 110 | hypothetical protein | - | - | - | - | - |
| 185 | - | ATG | 137021 137479 | 152 | hypothetical protein | - | - | - | - | - |
| 186 | - | ATG | 137489 137953 | 154 | hypothetical protein | - | - | - | - | - |
| 187 | - | ATG | 137958 138212 | 84 | hypothetical protein | - | - | - | - | - |
| 188 | - | ATG | 138209 138655 | 148 | Bacteriophage protein GP30.3 | [gp30.3 conserved hypothetical protein [Aeromonas phage Aeh1]](http://blast.ncbi.nlm.nih.gov/Blast.cgi#alnHdr_38640191) | NP-944147.1 | 54 | 91 | 3e -42 |
|  |  |  | 138675 138693 |  | terminator | - | - | - | - | - |
| 189 | - | ATG | 138714 139121 | 135 | hypothetical protein | [hypothetical protein VPHG-00060 [Vibrio phage 11895-B1]](http://blast.ncbi.nlm.nih.gov/Blast.cgi#alnHdr_472340018) | [YP-007673546.1](http://www.ncbi.nlm.nih.gov/protein/472340018?report=genbank&log$=prottop&blast_rank=1&RID=TACTG7FW014) | 53 | 94 | 5e -32 |
| 190 | - | ATG | 139215 139505 | 96 | hypothetical protein | - | - | - | - | - |
| 191 | - | ATG | 139537 141255 | 572 | hypothetical protein | [hypothetical protein VPHG-00061 [Vibrio phage 11895-B1]](http://blast.ncbi.nlm.nih.gov/Blast.cgi#alnHdr_472340019) | [YP-007673547.1](http://www.ncbi.nlm.nih.gov/protein/472340019?report=genbank&log$=prottop&blast_rank=1&RID=TAD4W3CR014) | 51 | 94 | 0 |
| 192 | - | ATG | 141330 141656 | 108 | hypothetical protein | [hypothetical protein Aeh1p254 [Aeromonas phage Aeh1]](http://blast.ncbi.nlm.nih.gov/Blast.cgi#alnHdr_38640176) | [NP-944132.1](http://www.ncbi.nlm.nih.gov/protein/38640176?report=genbank&log$=prottop&blast_rank=1&RID=TADFJ9FC014) | 37 | 93 | 1e -07 |
| 193 | - | ATG | 141672 142436 | 254 | ADP-ribosyltransferase | - | - | - | - | - |
|  |  |  | 142512 142531 |  | terminator | - | - | - | - | - |
| 194 | - | ATG | 142560 142877 | 105 | hypothetical protein | - | - | - | - | - |
| 195 | - | ATG | 142864 143097 | 77 | hypothetical protein | - | - | - | - | - |
| 196 | - | ATG | 143163 143537 | 124 | hypothetical protein | - | - | - | - | - |
| 197 | - | ATG | 143628 144350 | 240 | hypothetical protein | - | - | - | - | - |
| 198 | - | ATG | 144398 144694 | 98 | hypothetical protein | - | - | - | - | - |
| 199 | - | ATG | 144687 144953 | 88 | hypothetical protein | - | - | - | - | - |
| 200 | - | ATG | 144982 145374 | 130 | hypothetical protein | - | - | - | - | - |
| 201 | - | ATG | 145448 145654 | 68 | hypothetical protein | - | - | - | - | - |
|  |  |  | 145731 145750 |  | terminator | - | - | - | - | - |
| 202 | - | ATG | 145760 146335 | 191 | hypothetical protein | [hypothetical protein pVp-1-0108 [Vibrio phage pVp-1]](http://blast.ncbi.nlm.nih.gov/Blast.cgi#alnHdr_422937728) | [YP-007007931.1](http://www.ncbi.nlm.nih.gov/protein/422937728?report=genbank&log$=prottop&blast_rank=1&RID=TAE7ESC7014) | 51 | 92 | 3e -60 |
| 203 | - | ATG | 146347 147516 | 389 | hypothetical protein | [conserved uncharacterised protein [Salmonella phage ViI]](http://blast.ncbi.nlm.nih.gov/Blast.cgi#alnHdr_326804701) | [YP-004327572.1](http://www.ncbi.nlm.nih.gov/protein/326804701?report=genbank&log$=prottop&blast_rank=1&RID=TAEEUPB1014) | 35 | 84 | 1e -44 |
| 204 | - | ATG | 147579 147869 | 96 | hypothetical protein | - | - | - | - | - |
| 205 | - | ATG | 147957 148421 | 154 | hypothetical protein | - | - | - | - | - |
|  |  |  | 148491 148510 |  | terminator | - | - | - | - | - |
| 206 | - | ATG | 148518 150011 | 497 | nicotinamide phosphoribosyl transferase | [putative nicotinamide phosphoribosyl transferase [Aeromonas phage Aes012]](http://blast.ncbi.nlm.nih.gov/Blast.cgi#alnHdr_472438007) | [YP-007677787.1](http://www.ncbi.nlm.nih.gov/protein/472438007?report=genbank&log$=prottop&blast_rank=4&RID=TAEVB6Z5014) | 43 | 97 | 4e -125 |
|  |  |  | 150042 150061 |  | terminator | - | - | - | - | - |
| 207 | - | TTG | 150097 151131 | 344 | UV DNA damage endonuclease | [putative UV damage repair endonuclease [Aeromonas phage 65]](http://blast.ncbi.nlm.nih.gov/Blast.cgi#alnHdr_326536766) | [YP-004301197.1](http://www.ncbi.nlm.nih.gov/protein/326536766?report=genbank&log$=prottop&blast_rank=1&RID=TAEWMF10015) | 40 | 92 | 7e -69 |
| 208 | + | ATG | 151972 152250 | 92 | hypothetical protein | - | - | - | - | - |
|  |  |  | 152271 152292 |  | terminator | - | - | - | - | - |
| 209 | + | ATG | 152321 153298 | 325 | Type I restriction -modification system methyltransferase subunit | [putative methyltransferase [Massilia sp. LC238]](http://blast.ncbi.nlm.nih.gov/Blast.cgi#alnHdr_668953985) | [KFC72680.1](http://www.ncbi.nlm.nih.gov/protein/668953985?report=genbank&log$=prottop&blast_rank=7&RID=TAFBUAFU014) | 37 | 73 | 3e -22 |
| 210 | + | ATG | 153299 154783 | 494 | DNA ligase | [gp30 DNA ligase [Aeromonas phage Aeh1]](http://blast.ncbi.nlm.nih.gov/Blast.cgi#alnHdr_38640183) | [NP-944139.1](http://www.ncbi.nlm.nih.gov/protein/38640183?report=genbank&log$=prottop&blast_rank=2&RID=TAFEGF0T015) | 44 | 94 | 2e -124 |
|  |  |  | 154790 154808 |  | terminator |  |  |  |  |  |
| 211 | - | ATG | 154812 155795 | 327 | baseplate wedge subunit | [gp8 baseplate wedge subunit [Aeromonas phage 65]](http://blast.ncbi.nlm.nih.gov/Blast.cgi#alnHdr_326536507) | [YP-004300938.1](http://www.ncbi.nlm.nih.gov/protein/326536507?report=genbank&log$=prottop&blast_rank=1&RID=TAHFUXN1014) | 62 | 100 | 4e -157 |
| 212 | + | ATG | 155859 156158 | 99 | hypothetical protein | - | - | - | - | - |
| 213 | + | ATG | 156176 156487 | 103 | hypothetical protein | - | - | - | - | - |
| 214 | + | ATG | 156504 158747 | 747 | Molecular chaperone, HSP90 family | [-](http://blast.ncbi.nlm.nih.gov/Blast.cgi#alnHdr_38639924) | - | - | - | - |
| 215 | + | ATG | 158818 159768 | 316 | hypothetical protein | [rIIB protector from prophage-induced early lysis [Aeromonas phage PX29]](http://blast.ncbi.nlm.nih.gov/Blast.cgi#alnHdr_593774136) | [YP-009011759.1](http://www.ncbi.nlm.nih.gov/protein/593774136?report=genbank&log$=prottop&blast_rank=1&RID=TAHXH8UE014) | 36 | 93 | 8e -51 |
| 216 | + | ATG | 159862 160407 | 181 | SprT-like family protein | [-](http://blast.ncbi.nlm.nih.gov/Blast.cgi#alnHdr_769914589) | - | - | - | - |
| 217 | + | ATG | 160489 160728 | 79 | hypothetical protein | - | - | - | - | - |
| 218 | + | ATG | 160716 161168 | 150 | hypothetical protein | - | - | - | - | - |
| 219 | + | ATG | 161295 161903 | 202 | hypothetical protein | [hypothetical protein LPPPVgp01 [Listonella phage phiHSIC]](http://blast.ncbi.nlm.nih.gov/Blast.cgi#alnHdr_62362364) | [YP-224229.1](http://www.ncbi.nlm.nih.gov/protein/62362364?report=genbank&log$=prottop&blast_rank=1&RID=TAKR5V99015) | 60 | 98 | 3e -77 |
| 220 | + | TTG | 162010 163320 | 436 | DNA gyrase subunit A | [gp52 topoisomerase II medium subunit [Aeromonas phage PX29]](http://blast.ncbi.nlm.nih.gov/Blast.cgi#alnHdr_593774131) | [YP-009011754.1](http://www.ncbi.nlm.nih.gov/protein/593774131?report=genbank&log$=prottop&blast_rank=2&RID=TAKYTSJD015) | 43 | 98 | 1e -113 |
| 221 | + | ATG | 163331 163573 | 84 | hypothetical protein | - | - | - | - | - |
| 222 | + | ATG | 163639 163893 | 97 | hypothetical protein | - | - | - | - | - |
| 223 | + | ATG | 163919 164212 | 97 | Anti-Sigma Factor A | [anti-sigma70 protein [Aeromonas phage CC2]](http://blast.ncbi.nlm.nih.gov/Blast.cgi#alnHdr_423261841) | [YP-007010410.1](http://www.ncbi.nlm.nih.gov/protein/423261841?report=genbank&log$=prottop&blast_rank=4&RID=TAMC48BP015) | 47 | 75 | 1e -12 |
| 224 | + | ATG | 164214 164582 | 122 | hypothetical protein | [conserved hypothetical protein [Vibrio phage KVP40]](http://blast.ncbi.nlm.nih.gov/Blast.cgi#alnHdr_34419537) | [NP-899550.1](http://www.ncbi.nlm.nih.gov/protein/34419537?report=genbank&log$=prottop&blast_rank=2&RID=TAMU9P96014) | 56 | 96 | 7e -39 |
| 225 | + | ATG | 164586 165071 | 161 | hypothetical protein | - | - | - | - | - |
| 226 | + | ATG | 165073 166029 | 318 | hypothetical protein | [hypothetical protein CC2-184 [Aeromonas phage CC2]](http://blast.ncbi.nlm.nih.gov/Blast.cgi#alnHdr_423261775) | [YP-007010210.1](http://www.ncbi.nlm.nih.gov/protein/423261775?report=genbank&log$=prottop&blast_rank=1&RID=TAMWSRCF015) | 36 | 97 | 2e -59 |
| 227 | + | ATG | 166131 166688 | 185 | hypothetical protein | - | - | - | - | - |
| 228 | + | ATG | 166704 167249 | 181 | hypothetical protein | - | - | - | - | - |
| 229 | - | ATG | 167302 167499 | 65 | hypothetical protein | - | - | - | - | - |
|  |  |  | 167506 167525 |  | terminator | - | - | - | - | - |
| 230 | - | ATG | 167534 168451 | 305 | hypothetical protein | - | - | - | - | - |
| 231 | - | ATG | 168454 170229 | 591 | Phage tail repeat like protein | [putative tail fiber [Salinivibrio phage CW02]](http://blast.ncbi.nlm.nih.gov/Blast.cgi#alnHdr_423261944) | [YP-007010546.1](http://www.ncbi.nlm.nih.gov/protein/423261944?report=genbank&log$=prottop&blast_rank=1&RID=TANKFJEW014) | 54 | 92 | 1e -120 |
| 232 | - | ATG | 170192 171112 | 306 | hypothetical protein | - | - | - | - | - |
| 233 | - | ATG | 171122 171385 | 87 | hypothetical protein | - | - | - | - | - |
|  |  |  | 171430 171449 |  | terminator |  | - | - | - | - |
| 234 | - | ATG | 171471 171755 | 94 | hypothetical protein | - | - | - | - | - |
| 235 | - | ATG | 171767 172120 | 117 | Chaperonin 10 Kd subunit | [gp31 head assembly cochaperone with GroEL [Aeromonas phage Aeh1]](http://blast.ncbi.nlm.nih.gov/Blast.cgi#alnHdr_38640197) | [NP-944153.1](http://www.ncbi.nlm.nih.gov/protein/38640197?report=genbank&log$=prottop&blast_rank=2&RID=TAPHMAAE015) | 48 | 90 | 1e -23 |
|  |  |  | 172151 172172 |  | terminator | - | - | - | - | - |
| 236 | - | ATG | 172184 172723 | 179 | deoxycytidylate deaminase | [Cd dCMP deaminase [Aeromonas phage PX29]](http://blast.ncbi.nlm.nih.gov/Blast.cgi#alnHdr_593774066) | [YP-009011689.1](http://www.ncbi.nlm.nih.gov/protein/593774066?report=genbank&log$=prottop&blast_rank=3&RID=TAPV7VMD015) | 52 | 97 | 1e -52 |
| 237 | - | ATG | 172723 172992 | 87 | hypothetical protein | - | - | - | - | - |
| 238 | - | ATG | 172989 173282 | 97 | hypothetical protein | - | - | - | - | - |
| 239 | - | ATG | 173346 174293 | 315 | polynucleotide kinase | [polynucleotide 5'-kinase and 3'-phosphatase [Aeromonas phage CC2](http://blast.ncbi.nlm.nih.gov/Blast.cgi#alnHdr_423261779) | [YP-007010234.1](http://www.ncbi.nlm.nih.gov/protein/423261779?report=genbank&log$=prottop&blast_rank=1&RID=TAR261YS015) | 39 | 99 | 4e -58 |
| 240 | - | ATG | 174370 175500 | 376 | hypothetical protein | [RNA ligase [Caulobacter phage Cr30]](http://blast.ncbi.nlm.nih.gov/Blast.cgi#alnHdr_725949196) | [YP-009098789.1](http://www.ncbi.nlm.nih.gov/protein/725949196?report=genbank&log$=prottop&blast_rank=5&RID=TAR3CYNF014) | 40 | 98 | 5e -68 |
| 241 | - | ATG | 175628 176152 | 174 | hypothetical protein | [Vs.1 conserved transglycosylase SLT domain protein [Aeromonas phage 65]](http://blast.ncbi.nlm.nih.gov/Blast.cgi#alnHdr_326536824) | [YP-004301256.1](http://www.ncbi.nlm.nih.gov/protein/326536824?report=genbank&log$=prottop&blast_rank=1&RID=TASFJWWT014) | 39 | 96 | 5e -33 |
| 242 | - | ATG | 176156 176404 | 82 | hypothetical protein | - | - | - | - | - |
| 243 | + | ATG | 176569 177192 | 207 | hypothetical protein | - | - | - | - | - |
| 244 | + | ATG | 177206 177781 | 191 | hypothetical protein | - | - | - | - | - |
| 245 | + | ATG | 177796 178500 | 234 | hypothetical protein | - | - | - | - |  |
| 246 | + | ATG | 178561 178830 | 89 | hypothetical protein | - | - | - | - | - |
| 247 | - | ATG | 178819 179589 | 256 | neck protein | [neck protein [Citrobacter phage Moon]](http://blast.ncbi.nlm.nih.gov/Blast.cgi#alnHdr_723006415) | [AIX12152.1](http://www.ncbi.nlm.nih.gov/protein/723006415?report=genbank&log$=prottop&blast_rank=4&RID=TATET7NB015) | 50 | 83 | 2e -63 |
| 248 | - | ATG | 179593 180507 | 304 | neck protein | [gp13 neck protein [Aeromonas phage 65]](http://blast.ncbi.nlm.nih.gov/Blast.cgi#alnHdr_326536501) | [YP-004300932.1](http://www.ncbi.nlm.nih.gov/protein/326536501?report=genbank&log$=prottop&blast_rank=3&RID=TATFWSYW014) | 50 | 100 | 2e -102 |
|  |  |  | 180541 180560 |  | terminator | - | - | - | - | - |
| 249 | - | ATG | 180567 180743 | 58 | hypothetical protein | - | - | - | - |  |
| 250 | - | ATG | 180764 182014 | 416 | hypothetical protein | **-** | - | - | - | - |
| 251 | - | ATG | 182031 185402 | 1123 | fibritin | [putative fibritin neck whiskers [Cronobacter phage S13]](http://blast.ncbi.nlm.nih.gov/Blast.cgi#alnHdr_641468790) | [AIA64894.1](http://www.ncbi.nlm.nih.gov/protein/641468790?report=genbank&log$=prottop&blast_rank=1&RID=TAUB6T54014) | 36 | 76 | 2e -38 |
|  |  |  | 185451 185467 |  | terminator | - | - | - | - | - |
| 252 | - | ATG | 185474 186820 | 448 | long tail fiber, proximal subunit | [gp12 short tail fibers [Aeromonas phage Aeh1]](http://blast.ncbi.nlm.nih.gov/Blast.cgi#alnHdr_38640138) | NP-944094.1 | 42 | 74 | 3e -55 |
| 253 | - | ATG | 186820 187773 | 317 | baseplate wedge subunit and tail pin | [baseplate wedge subunit and tail pin [Aeromonas phage CC2]](http://blast.ncbi.nlm.nih.gov/Blast.cgi#alnHdr_423261821) | [YP-007010352.1](http://www.ncbi.nlm.nih.gov/protein/423261821?report=genbank&log$=prottop&blast_rank=1&RID=TAUVP2S2014) | 37 | 99 | 1e -24 |
| 254 | - | ATG | 187776 189917 | 713 | baseplate wedge subunit and tail pin | [gp10 baseplate wedge subunit and tail pin [Aeromonas phage PX29]](http://blast.ncbi.nlm.nih.gov/Blast.cgi#alnHdr_593774004) | [YP-009011627.1](http://www.ncbi.nlm.nih.gov/protein/593774004?report=genbank&log$=prottop&blast_rank=1&RID=TAV59ZWZ015) | 50 | 99 | 0 |
| 255 | - | ATG | 189929 190852 | 307 | baseplate wedge tail fiber connector | [baseplate wedge tail fiber connector [Aeromonas phage CC2]](http://blast.ncbi.nlm.nih.gov/Blast.cgi#alnHdr_423261823) | [YP-007010354.1](http://www.ncbi.nlm.nih.gov/protein/423261823?report=genbank&log$=prottop&blast_rank=1&RID=TAVCT9SY014) | 51 | 99 | 3e -105 |
|  |  |  | 190893 190911 |  | terminator | - | - | - | - | - |
| 256 | - | ATG | 190935 191666 | 243 | Modification methylase DpnIIB | [DNA (cytosine-5-)-methyltransferase [Firmicutes bacterium CAG:238]](http://blast.ncbi.nlm.nih.gov/Blast.cgi#alnHdr_547732256) | [WP-022146239.1](http://www.ncbi.nlm.nih.gov/protein/547732256?report=genbank&log$=prottop&blast_rank=1&RID=TAVDXKRR014) | 40 | 91 | 8e -52 |
| 257 | - | ATG | 191654 192343 | 229 | Serine/threonine-protein phosphatase 1 | [serine/threonine-protein phosphatase 1 [Vibrio phage pYD21-A]](http://blast.ncbi.nlm.nih.gov/Blast.cgi#alnHdr_472340446) | [YP-007673971.1](http://www.ncbi.nlm.nih.gov/protein/472340446?report=genbank&log$=prottop&blast_rank=1&RID=TAVSV3D9014) | 52 | 96 | 3e -69 |
|  |  |  | 192395 192412 |  | terminator | - | - | - | - | - |
| 258 | - | ATG | 192423 195752 | 1109 | baseplate wedge subunit | [gp7 baseplate wedge initiator [Aeromonas phage Aeh1]](http://blast.ncbi.nlm.nih.gov/Blast.cgi#alnHdr_38640133) | [NP-944089.1](http://www.ncbi.nlm.nih.gov/protein/38640133?report=genbank&log$=prottop&blast_rank=1&RID=TAVUS0T6015) | 48 | 99 | 0 |
|  |  |  | 195805 195823 |  | terminator | - | - | - | - | - |
| 259 | - | ATG | 195832 196119 | 95 | hypothetical protein | [hypothetical protein CC2-334 [Aeromonas phage CC2]](http://blast.ncbi.nlm.nih.gov/Blast.cgi#alnHdr_423261752) | [YP-007010360.1](http://www.ncbi.nlm.nih.gov/protein/423261752?report=genbank&log$=prottop&blast_rank=2&RID=TAWJZK5X014) | 52 | 100 | 1e -21 |
| 260 | - | ATG | 196132 196686 | 184 | hypothetical protein | [gp5.1 conserved hypothetical protein [Aeromonas phage Aeh1]](http://blast.ncbi.nlm.nih.gov/Blast.cgi#alnHdr_38640129) | NP-944085.1 | 36 | 91 | 2e -16 |
| 261 | - | ATG | 196689 196874 | 61 | hypothetical protein | - | - | - | - | - |
| 262 | - | ATG | 196887 198692 | 601 | baseplate hub subunit and tail lysozyme | [gp5 baseplate hub subunit and tail lysozyme [Aeromonas phage Aeh1]](http://blast.ncbi.nlm.nih.gov/Blast.cgi#alnHdr_38640128) | [NP-944084.1](http://www.ncbi.nlm.nih.gov/protein/38640128?report=genbank&log$=prottop&blast_rank=2&RID=TAX219F4015) | 36 | 98 | 4e -73 |
| 263 | - | ATG | 198685 199899 | 404 | hypothetical protein | [hypothetical protein Aeh1p205 [Aeromonas phage Aeh1]](http://blast.ncbi.nlm.nih.gov/Blast.cgi#alnHdr_38640127) | [NP-944083.1](http://www.ncbi.nlm.nih.gov/protein/38640127?report=genbank&log$=prottop&blast_rank=1&RID=TAX3K439014) | 37 | 95 | 6e -43 |
