# Supplementary material for: Isolation, characterization and analysis of bacteriophages from the haloalkaline lake Elmenteita, Kenya
Source: PLoS One. 2019 Apr 25;14(4):e0215734. doi: 10.1371/journal.pone.0215734 (PMC6483233; doi:10.1371/journal.pone.0215734)
Supplement: S5 Table — ORFs are arranged according to their position (Start-End) in the genome. Significant database matches are given in the column marked Putative homolog. Tools used to search for similarity are blastn (nucleotide Blast search) or blastp (protein Blast search). Scores and E-values obtained in the Blast searches are given in the last three columns. Homology assignments were accepted only if the statistical significance of the sequence similarities (E value) was less than 1x10^-5^, the percentage query cover was ≥60% and the percentage identity between the aligned sequences was ≥35%. (DOCX) [file pone.0215734.s006.docx]

**Supplementary Table 5:** Overview of bacteriophage vB-EauS-123 ORFs and summary of homology searches.

| **ORF No.** | **Strand** | **Start Codon** | **Start End** | **aa** | **Function** | **Putative homolog** | **Accession** | **% Identity** | **% Query cover** | **E-Value** |
| --- | --- | --- | --- | --- | --- | --- | --- | --- | --- | --- |
| 1 | - | ATG | 9 230 | 73 | hypothetical protein | - | - | - | - | - |
| 2 | - | ATG | 227 2491 | 754 | phage tail tape measure protein, TP901 family, core region | [phage tail tape measure protein [Lactobacillus ceti]](http://blast.ncbi.nlm.nih.gov/Blast.cgi#alnHdr_652816747) | WP-027107145.1 | 43 | 91 | 1e-152 |
|  |  | ATG | 2673 2708 |  | terminator | - | - | - | - | - |
| 3 | - | ATG | 2729 3076 | 115 | hypothetical protein | [hypothetical protein GPGV1-gp27 [Geobacillus phage GBSV1]](http://blast.ncbi.nlm.nih.gov/Blast.cgi#alnHdr_115334637) | YP-764483.1 | 50 | 99 | 3e-35 |
| 4 | - | ATG | 3080 3649 | 104 | phage major tail protein, phi13 family | [phage protein [Jeotgalibacillus sp. D5]](http://blast.ncbi.nlm.nih.gov/Blast.cgi#alnHdr_748255554) | WP-039810911.1 | 62 | 99 | 9e-36 |
| 5 | - | ATG | 3651 3965 | 120 | hypothetical protein | prophage pi2 protein 37 [Streptococcus pseudopneumoniae] | WP-049543884.1 | 50 | 93 | 2e-31 |
| 6 | - | ATG | 3962 4324 | 102 | hypothetical protein | [hypothetical protein GPGV1-gp23 [Geobacillus phage GBSV1]](http://blast.ncbi.nlm.nih.gov/Blast.cgi#alnHdr_115334633) | YP-764479.1 | 54 | 99 | 2e-29 |
| 7 | - | ATG | 4317 4625 | 103 | hypothetical protein | hypothetical protein GPGV1-gp22 [Geobacillus phage GBSV1] | YP-764478.1 | 58 | 88 | 5e-27 |
| 8 | - | ATG | 4618 4929 | 57 | hypothetical protein | - | - | - | - | - |
| 9 | - | ATG | 4922 5095 | 428 | phage major capsid protein, HK97 family | phage prohead [Geobacillus phage GBSV1] | YP-764475.1 | 59 | 96 | 2e-68 |
| 10 | - | GTG | 5137 6423 | 191 | phage prohead protease, HK97 family | [phage portal protein [Geobacillus phage GBSV1]](http://blast.ncbi.nlm.nih.gov/Blast.cgi#alnHdr_155042935) | YP-764474.1 | 60 | 99 | 1e-172 |
| 11 | - | ATG | 6414 6989 | 391 | phage portal protein, HK97 family | phage terminase [Geobacillus phage GBSV1] | YP-764473.1 | 72 | 99 | 0 |
| 12 | - | ATG | 6986 8161 | 573 | Phage terminase-like protein, large subunit | terminase [Paenibacillus polymyxa] | WP-025364415.1 | 65 | 97 | 1e-42 |
| 13 | - | ATG | 8186 9907 | 113 | Phage terminase, small subunit | phage-related terminase protein small subunit, putative [Listeria welshimeri serovar 6b str. SLCC5334] | CAK20639.1 | 59 | 88 | 1e-32 |
| 14 | - | ATG | 9916 10257 | 70 | hypothetical protein | - | - | - | - | - |
|  |  |  | 10926 10951 |  | terminator |  |  |  |  |  |
| 15 | - | ATG | 10686 10898 | 141 | hypothetical protein | - | - | - | - | - |
| 16 | - | ATG | 11026 11451 | 131 | hypothetical protein | - | - | - | - | - |
| 17 | - | ATG | 11456 11851 | 60 | hypothetical protein | - | - | - | - | - |
| 18 | - | ATG | 11838 12020 | 57 | hypothetical protein | - | - | - | - | - |
| 19 | - | ATG | 12017 12190 | 34 | hypothetical protein | - | - | - | - | - |
| 20 | - | ATG | 12183 12287 | 65 | hypothetical protein | - | - | - | - | - |
| 21 | - | ATG | 12305 12502 | 82 | hypothetical protein | - | - | - | - | - |
| 22 | - | ATG | 12529 12777 | 91 | hypothetical protein | - | - | - | - | - |
| 23 | - | ATG | 12797 13072 | 121 | hypothetical protein | - | - | - | - | - |
| 24 | - | ATG | 13126 13491 | 103 | hypothetical protein | - | - | - | - | - |
| 25 | - | ATG | 13484 13795 | 63 | hypothetical protein | - | - | - | - | - |
| 26 | - | ATG | 13785 13976 | 75 | hypothetical protein | - | - | - | - | - |
| 27 | - | ATG | 13973 14200 | 182 | dUTPase | dUTPase [Bacillus phage Stills] | AKC02653.1 | 56 | 98 | 1e-53 |
| 28 | - | ATG | 14203 14751 | 76 | hypothetical protein | - | - | - | - | - |
| 29 | - | ATG | 14748 14978 | 36 | hypothetical protein | - | - | - | - | - |
| 30 | - | ATG | 14975 15085 | 35 | hypothetical protein | - | - | - | - | - |
| 31 | - | ATG | 15177 15284 | 67 | hypothetical protein | - | - | - | - | - |
| 32 | - | ATG | 15303 15506 | 71 | hypothetical protein | - | - | - | - | - |
| 33 | - | ATG | 15503 15718 | 60 | hypothetical protein | - | - | - | - | - |
| 34 | - | ATG | 15715 15897 | 73 | hypothetical protein | - | - | - | - | - |
| 35 | - | ATG | 15902 16123 | 138 | hypothetical protein | Endodeoxyribonuclease RusA [Bacillus cereus] | CUB08538.1 | 40 | 96 | 2e-24 |
| 36 | - | ATG | 16256 16672 | 188 | Holliday junction resolvase | - | - | - | - | - |
| 37 | - | ATG | 16669 17235 | 83 | hypothetical protein | - | - | - | - | - |
| 38 | - | ATG | 17232 17483 | 70 | hypothetical protein | - | - | - | - | - |
| 39 | - | ATG | 17506 17718 | 218 | phage regulatory protein, Rha family | rha family phage regulatory protein [Enterococcus faecalis] | WP-010815797.1 | 41 | 99 | 3e-45 |
| 40 | - | ATG | 17727 18383 | 54 | hypothetical protein | - | - | - | - | - |
|  |  |  | 18486 18509 |  | terminator |  |  |  |  |  |
| 41 | - | ATG | 18483 18647 | 74 | hypothetical protein | - | - | - | - | - |
| 42 | - | ATG | 18644 18868 | 85 | hypothetical protein | - | - | - | - | - |
| 43 | - | ATG | 18865 19122 | 265 | DNA replication protein DnaC | DnaC replication protein [Virgibacillus sp. Vm-5] | WP-051739281.1 | 40 | 89 | 2e-48 |
| 44 | - | ATG | 19136 19933 | 287 | DnaD domain protein | - | - | - | - | - |
| 45 | - | ATG | 19953 20816 | 46 | hypothetical protein | - | - | - | - | - |
| 46 | - | ATG | 20989 21198 | 69 | Helix-turn-helix | XRE family transcriptional regulator [Bordetella petrii] | WP-026638648.1 | 40 | 75 | 0.016 |
| 47 | + | GTG | 21331 21693 | 120 | HTH-type transcriptional regulator ImmR | [XRE family transcriptional regulator [Desulfosporosinus meridiei]](http://blast.ncbi.nlm.nih.gov/Blast.cgi#alnHdr_504718098) | WP-014905200.1 | 40 | 94 | 1e-16 |
| 48 | + | ATG | 21710 22126 | 138 | hypothetical protein | - | - | - | - | - |
| 49 | + | ATG | 22126 23673 | 515 | Recombinase | recombinase [Geobacillus sp. T6] | WP-047752244.1 | 38 | 95 | 2e-96 |
|  |  |  | 23780 23810 |  | terminator | - | - | - | - | - |
|  |  |  | 23850 23869 |  | terminator | - | - | - | - | - |
| 50 | - | ATG | 24491 24736 | 81 | holin, SPP1 family | holin [Staphylococcus haemolyticus] | WP-053040140.1 | 45 | 91 | 6e-11 |
| 51 | - | TTG | 24738 25508 | 256 | Sporulation-specific N-acetylmuramoyl-L-alanine amidase | [N-acetylmuramoyl-L-alanine amidase [Bacillus clausii]](http://blast.ncbi.nlm.nih.gov/Blast.cgi#alnHdr_499566874) | WP-011247657.1 | 53 | 71 | 8e-54 |
| 52 | - | ATG | *25521 25913 | 130 | hypothetical protein | hypothetical protein [Exiguobacterium sp. AB2] | WP-034806723.1 | 43 | 87 | 3e-17 |
|  |  |  | 26146 26163 |  | terminator | - | - | - | - | - |
| 53 | - | ATG | 26211 28337 | 708 | hypothetical protein | - | - | - | - | - |
| 54 | - | ATG | 28356 30317 | 653 | hypothetical protein | [putative minor structural protein [Bacillus sp. LF1]](http://blast.ncbi.nlm.nih.gov/Blast.cgi#alnHdr_857575752) | CRK80298.1 | 47 | 66 | 4e-127 |
| 55 | - | ATG | 30330 30923 | 110 | Phage tail protein | phage tail protein [Bacillus amyloliquefaciens] |  | 47 | 98 | 8e-22 |

* Transcriptional start site
