# Supplementary material for: Isolation, characterization and analysis of bacteriophages from the haloalkaline lake Elmenteita, Kenya
Source: PLoS One. 2019 Apr 25;14(4):e0215734. doi: 10.1371/journal.pone.0215734 (PMC6483233; doi:10.1371/journal.pone.0215734)
Supplement: S6 Table — ORFs are arranged according to their position (Start-End) in the genome. Significant database matches are given in the column marked Putative homolog. Tools used to search for similarity are blastn (nucleotide Blast search) or blastp (protein Blast search). Scores and E-values obtained in the Blast searches are given in the last three columns. Homology assignments were accepted only if the statistical significance of the sequence similarities (E value) was less than 1x10^-5^, the percentage query cover was ≥60% and the percentage identity between the aligned sequences was ≥35%. (DOCX) [file pone.0215734.s007.docx]

**Supplementary Table 6:** Overview of bacteriophage vB-BhaS-171 ORFs and summary of homology searches.

| **ORF No.** | **Strand** | **Start codon** | **Start End** | **aa** | **Function** | **Putative homolog** | **Accession** | **% Identity** | **% Query cover** | **E-Value** |
| --- | --- | --- | --- | --- | --- | --- | --- | --- | --- | --- |
| 1 | - | ATG | 50 454 | 134 | Single-stranded DNA-binding protein SsbB | [single-stranded DNA-binding protein [Oceanobacillus sp. S5]](http://blast.ncbi.nlm.nih.gov/Blast.cgi#alnHdr_751272013) | WP-040980887.1 | 41 | 100 | 6e-26 |
| 2 | - | ATG | 451 1260 | 269 | Modification methylase HhaI | DNA (cytosine-5-)-methyltransferase [Clostridium sp. ASF356] | WP-004036918.1 | 49 | 98 | 2e-78 |
| 3 | - | ATG | 1481 1633 | 50 | hypothetical protein | - | - | - | - | - |
| 4 | - | ATG | 1665 1826 | 53 | hypothetical protein | - | - | - | - | - |
| 5 | - | ATG | 1849 2364 | 171 | putative homing endonuclease | [HNH endonuclease [Geobacillus sp. 12AMOR1]](http://blast.ncbi.nlm.nih.gov/Blast.cgi#alnHdr_836671447) | WP-047819363.1 | 45 | 74 | 4e-27 |
| 6 | - | ATG | 2364 2693 | 109 | hypothetical protein | - | - | - | - | - |
| 7 | - | ATG | 2690 2932 | 80 | hypothetical protein | [phage-related protein [Bacillus clausii KSM-K16]](http://blast.ncbi.nlm.nih.gov/Blast.cgi#alnHdr_56909384) | BAD63911.1 | 51 | 85 | 1e-10 |
| 8 | - | ATG | 2929 3126 | 65 | hypothetical protein | hypothetical protein GBVE2-gp041 [Geobacillus virus E2] | YP-001285847.1 | 47 | 98 | 9e-11 |
| 9 | - | GTG | 3126 4400 | 424 | Replicative DNA helicase | Replicative DNA helicase [Bacillus cereus ATCC 10876] | EEK49359.1 | 42 | 99 | 3e-101 |
| 10 | - | TTG | 4401 5126 | 241 | hypothetical protein | [hypothetical phage-related protein [Staphylococcus aureus]](http://blast.ncbi.nlm.nih.gov/Blast.cgi#alnHdr_678273684) | CDR28674.1 | 46 | 99 | 4e-62 |
| 11 | - | TTG | 5223 5369 | 48 | hypothetical protein | - | - | - | - | - |
| 12 | + | ATG | 5582 5752 | 56 | hypothetical protein | [hypothetical protein phiCD27-gp47 [Clostridium phage phiCD27]](http://blast.ncbi.nlm.nih.gov/Blast.cgi#alnHdr_209901284) | YP-002290923.1 | 46 | 73 | 0.002 |
|  |  |  | 5741 5761 |  | terminator |  |  |  |  |  |
| 13 | - | ATG | 5783 6343 | 186 | hypothetical protein | - | - | - | - | - |
| 14 | - | ATG | 6377 6574 | 65 | hypothetical protein | - | - | - | - | - |
| 15 | - | ATG | 6618 6842 | 74 | hypothetical protein | - | - | - | - | - |
| 16 | - | ATG | 6839 7159 | 106 | hypothetical protein | - | - | - | - | - |
| 17 | - | ATG | 7156 7413 | 85 | hypothetical protein | - | - | - | - | - |
| 18 | - | TTG | 7438 7968 | 76 | hypothetical protein | - | - | - | - | - |
| 19 | - | ATG | 8039 8215 | 58 | hypothetical protein | - | - | - | - | - |
| 20 | - | ATG | 8163 8423 | 86 | hypothetical protein | - | - | - | - | - |
| 21 | - | ATG | 8407 8805 | 132 | hypothetical protein | - | - | - | - | - |
| 22 | - | ATG | 8847 9083 | 78 | hypothetical protein | - | - | - | - | - |
| 23 | - | ATG | 9127 9300 | 57 | Helix-turn-helix domain protein | Prophage LambdaBa02, DNA-binding protein [Bacillus thuringiensis MC28] | AFU14622.1 | 50 | 87 | 4e-10 |
| 24 | - | TTG | 9337 9519 | 60 | conjugal transfer protein TrbA | helix-turn-helix/TPR domain-containing protein [Ornithinibacillus scapharcae] | WP-010099525.1 | 52 | 100 | 3e-13 |
| 25 | + | ATG | 9720 10067 | 115 | HTH-type transcriptional regulator PuuR | hypothetical protein [Virgibacillus sp. Vm-5] |  | 47 | 95 | 1e-24 |
| 26 | + | ATG | 10208 10312 | 34 | hypothetical protein | - | - | - | - | - |
| 27 | + | ATG | 10391 11452 | 353 | Integrase |  | WP-000323190.1 | 40 | 99 | 2e-78 |
|  |  | ATG | 11782 11806 |  | terminator | - | - | - | - | - |
| 28 | - | TTG | 11791 12399 | 202 | hypothetical protein | hypothetical protein [Bacillus phage PM1] | YP-007678110.1 | 43 | 95 | 3e-44 |
| 29 | - | TTG | 12338 13564 | 408 | DNA translocase FtsK | FtsK/SpoIIIE family protein [Bacillus virus 1] | YP-001522914.1 | 40 | 93 | 1e-91 |
| 30 | - | ATG | 13802 14077 | 91 | hypothetical protein | - | - | - | - | - |
| 31 | - | ATG | 14074 14238 | 54 | hypothetical protein | - | - | - | - | - |
| 32 | + | ATG | 14388 14597 | 69 | Helix-turn-helix domain protein | [Helix-turn-helix [Bacillus subtilis]](http://blast.ncbi.nlm.nih.gov/Blast.cgi#alnHdr_924093376) | CUB59637.1 | 47 | 97 | 1e-12 |
|  |  | ATG | 14587 14604 |  | terminator | - | - | - | - | - |
| 33 | - | ATG | 14605 15333 | 242 | N-acetylmuramoyl-L-alanine amidase | [N-acetylmuramoyl-L-alanine amidase domain-containing protein [Listeria phage LP-030-3]](http://blast.ncbi.nlm.nih.gov/Blast.cgi#alnHdr_658607684) | YP-009044695.1 | 44 | 76 | 2e-41 |
| 34 | - | ATG | 15333 15599 | 88 | Phage holin | [holin [Bacillus alcalophilus]](http://blast.ncbi.nlm.nih.gov/Blast.cgi#alnHdr_490562995) | WP-004428037.1 | 61 | 98 | 1e-33 |
| 35 | - | TTG | 15611 15925 | 104 | hypothetical protein | [hypothetical protein BCBBV1cgp56 [Bacillus phage BCJA1c]](http://blast.ncbi.nlm.nih.gov/Blast.cgi#alnHdr_56694924) | YP-164434.1 | 65 | 95 | 2e-37 |
| 36 | - | TTG | 15978 16295 | 105 | hypothetical protein | - | - | - | - | - |
| 37 | - | ATG | 16353 16550 | 65 | hypothetical protein | [hypothetical protein [Bacillus panaciterrae]](http://blast.ncbi.nlm.nih.gov/Blast.cgi#alnHdr_736663063) | WP-034670105.1 | 47 | 84 | 1e-07 |
| 38 | - | ATG | 16561 18456 | 631 | hypothetical protein | phage minor structural protein [Bacillus marisflavi] | WP-048005997.1 | 52 | 99 | 0 |
| 39 | - | ATG | 18465 20429 | 654 | Pectate lyase superfamily protein | hypothetical protein [Bacillus sp. FJAT-27245] | WP-053365785.1 | 43 | 93 | 3e-147 |
|  |  | ATG | 20455 20474 |  | terminator | - | - | - | - | - |
| 40 | - | ATG | 20526 20666 | 46 | hypothetical protein | - | - | - | - | - |
| 41 | - | ATG | 20698 20892 | 64 | hypothetical protein | - | - | - | - | - |
| 42 | - | GTG | *20929 22743 | 604 | GDSL-like Lipase/Acylhydrolase | [phage pre-neck appendage-like protein [Clostridium botulinum V891]](http://blast.ncbi.nlm.nih.gov/Blast.cgi#alnHdr_835630761) | KLU74191.1 | 43 | 61 | 1e-86 |
| 43 | - | ATG | 22759 23622 | 287 | Phage-related protein | - | - | - | - | - |
| 44 | - | GTG | 23619 25703 | 694 | phage tail tape measure protein, TP901 family, core region | phage tail tape measure protein [Oscillibacter valericigenes] | WP-014118137.1 | 48 | 47 | 1e-83 |
| 45 | - | GTG | 25681 25920 | 79 | hypothetical protein | - | - | - | - | - |
| 46 | - | ATG | 25989 26294 | 101 | hypothetical protein | hypothetical protein [Pseudobacteroides cellulosolvens] | WP-036939439.1 | 54 | 97 | 9e-27 |
|  |  |  | 26322 26341 |  | terminator | - | - | - | - | - |
| 47 | - | ATG | 26381 26965 | 194 | hypothetical protein | [hypothetical protein [Paenibacillus sp. FSL P4-0081]](http://blast.ncbi.nlm.nih.gov/Blast.cgi#alnHdr_918369338) | WP-052421613.1 | 56 | 98 | 3e-73 |
| 48 | - | ATG | 26970 27332 | 120 | hypothetical protein | [hypothetical protein [Pseudobacteroides cellulosolvens]](http://blast.ncbi.nlm.nih.gov/Blast.cgi#alnHdr_739068033) | WP-036939435.1 | 40 | 96 | 2e-22 |
| 49 | - | ATG | 27329 27778 | 149 | phage protein, HK97 gp10 family | [hypothetical protein [Desulfosporosinus sp. OT]](http://blast.ncbi.nlm.nih.gov/Blast.cgi#alnHdr_750108745) | WP-040413428.1 | 62 | 100 | 3e-63 |
| 50 | - | ATG | 27779 28126 | 115 | putative phage head-tail adaptor | [head-tail adaptor protein [Bacillus alveayuensis]](http://blast.ncbi.nlm.nih.gov/Blast.cgi#alnHdr_768724433) | WP-044893571.1 | 57 | 100 | 6e-42 |
| 51 | - | ATG | 28113 28415 | 100 | hypothetical protein | gp7 [Bacillus sp. LF1] | CRK80309.1 | 84 | 88 | 2e-44 |
| 52 | - | ATG | 28421 28606 | 61 | hypothetical protein | [hypothetical protein [Anaerobacillus macyae]](http://blast.ncbi.nlm.nih.gov/Blast.cgi#alnHdr_852223318) | WP-048309719.1 | 59 | 88 | 1e-13 |
| 53 | - | ATG | 28627 29766 | 379 | phage major capsid protein, HK97 family | phage capsid protein [Bacillus sp. FJAT-13831] | WP-017154127.1 | 81 | 100 | 0 |
| 54 | - | GTG | 29783 30541 | 252 | ATP-dependent Clp protease proteolytic subunit 1 | ATP-dependent Clp protease proteolytic subunit 1 [Planomicrobium sp. ES2] | CEG23133.1 | 71 | 98 | 1e-128 |
| 55 | - | TTG | 30538 31674 | 378 | phage portal protein, HK97 family | HK97 family phage portal protein [Bacillus cereus CER057] | EJQ59179.1 | 73 | 99 | 0 |
| 56 | - | ATG | 31690 33324 | 544 | Phage terminase-like protein, large subunit | phage terminase large subunit [Paenibacillus sp. JCM 10914] | WP-023966576.1 | 86 | 98 | 0 |
| 57 | - | ATG | 33321 33629 | 102 | putative phage terminase, small subunit, P27 family | Phage terminase, small subunit [Halobacillus dabanensis] | CDQ17952.1 | 60 | 98 | 1e-32 |
| 58 | - | ATG | 33736 34065 | 109 | hypothetical protein | HNH endonuclease [Bacillus subtilis] | CUB58140.1 | 61 | 100 | 2e-40 |
| 59 | - | ATG | 34094 34255 | 53 | hypothetical protein | - | - | - | - | - |
| 60 | - | ATG | 34309 34998 | 229 | hypothetical protein | [hypothetical protein 39-O-gp07 [Clostridium phage phiCP39-O]](http://blast.ncbi.nlm.nih.gov/Blast.cgi#alnHdr_208429862) | YP-002265415.1 | 45 | 73 | 7e-42 |
| 61 | - | ATG | 35113 36387 | 424 | hypothetical protein | [gp163 [Sphingomonas phage PAU]](http://blast.ncbi.nlm.nih.gov/Blast.cgi#alnHdr_435844666) | YP-007006770.1 | 40 | 60 | 9e-44 |
| 62 | - | ATG | 36752 36913 | 53 | hypothetical protein | - | - | - | - | - |
| 63 | - | ATG | 36942 37358 | 138 | hypothetical protein | - | - | - | - | - |
| 64 | - | GTG | 37359 37787 | 142 | hypothetical protein | [hypothetical protein GBVE2-gp055 [Geobacillus virus E2]](http://blast.ncbi.nlm.nih.gov/Blast.cgi#alnHdr_148747782) | YP-001285861.1 | 58 | 96 | 6e-50 |
| 65 | - | GTG | 37784 37918 | 44 | hypothetical protein | - | - | - | - | - |
| 66 | - | GTG | 37911 38477 | 188 | hypothetical protein | [protein, Lmo2306 homolog [Bacteriophage A118] [Bacillus sp. GeD10]](http://blast.ncbi.nlm.nih.gov/Blast.cgi#alnHdr_493975009) | WP-006918081.1 | 51 | 70 | 2e-35 |
| 67 | - | ATG | 38446 38772 | 108 | MazG nucleotide pyrophosphohydrolase domain protein | MazG nucleotide pyrophosphohydrolase [Clostridiales bacterium oral taxon 876] | WP-021653377.1 | 50 | 99 | 1e-32 |

* Transcriptional start site
